# Repression of productive viral replication by KSHV LANA correlates with reduced adaptive immune activation: potential implications for KSHV immune evasion

**DOI:** 10.1128/mbio.00297-26

**Published:** 2026-07-23

**Authors:** Steven J. Murdock, Shana M. Owens, Darby G. Oldenburg, J. Craig Forrest

**Affiliations:** 1 Department of Microbiology and Immunology, University of Arkansas for Medical Sciences 12215, Little Rock, Arkansas, USA; 2 Gundersen Lutheran Medical Foundation 543836, La Crosse, Wisconsin, USA; 3 Center for Microbial Pathogenesis and Host Inflammatory Responses, University of Arkansas for Medical Sciences 12215, Little Rock, Arkansas, USA; 4 Winthrop P. Rockefeller Cancer Institute, University of Arkansas for Medical Sciences 12215, Little Rock, Arkansas, USA; The University of North Carolina at Chapel Hill, Chapel Hill, North Carolina, USA

**Keywords:** murine gammaherpesvirus 68, Kaposi's sarcoma-associated herpesvirus, latency-associated nuclear antigen (LANA), adaptive immunity, oncogenic virus, viral replication, viral pathogenesis, immune evasion

## Abstract

Adaptive immune responses to primary Kaposi sarcoma-associated herpesvirus (KSHV) infection are poorly defined. To develop better small-animal models for understanding KSHV pathogenesis and immunity, we previously generated a chimeric virus in which the KSHV latency-associated nuclear antigen (kLANA), a conserved multifunctional protein critical for viral latency, was exchanged for the LANA homolog in murine gammaherpesvirus 68 (MHV68). Despite supporting comparable levels of latent infection between wild-type (WT) and KLKI MHV68, kLANA directly repressed MHV68 lytic replication and reactivation. We therefore hypothesized that suppression of lytic replication by kLANA dampens adaptive immune responses. To test this, mice were infected with equivalent doses of either WT or KLKI MHV68, and adaptive immune responses were evaluated over time. Compared to the WT virus, polyclonal B and T cell activation was starkly reduced following KLKI MHV68 infection, which correlated with reduced virus-specific humoral immunity and effector CD4^+^ and CD8^+^ T cell activation. Immune activation phenotypes were independent of the inoculating dose, as a high-dose infection with KLKI MHV68 still resulted in comparatively reduced adaptive immune activation. In contrast, infection of *Ifnar1^−/−^* mice, which support enhanced KLKI MHV68 lytic replication, led to potent adaptive cellular and humoral immune activation by both WT and KLKI viruses, suggesting that a lytic replication threshold must be passed for viral antigen-driven adaptive immune engagement. Collectively, these data support the hypothesis that kLANA-mediated suppression of lytic replication limits polyclonal lymphocyte activation and facilitates adaptive immune evasion by holding viral replication below an antigenic activation threshold.

**IMPORTANCE:**

KSHV is a gammaherpesvirus that establishes lifelong, chronic infections in humans and increases the risk of virus-associated cancers. Currently, there is little information on how primary KSHV infection influences adaptive immune development in healthy individuals. Rodent models, such as murine gammaherpesvirus 68 (MHV68), provide a valuable laboratory system for studying gammaherpesvirus pathogenesis *in vivo*. In this study, we report that infection with a previously characterized chimeric KSHV-MHV68 virus expressing KSHV LANA represses lytic viral replication and elicits weak adaptive immune responses following primary infection, despite efficient latency establishment. By enhancing KLKI MHV68 replication *in vivo*, we restore adaptive immune activation, providing evidence that a viral replication threshold must be eclipsed for potent adaptive immune engagement. We propose that KSHV, through LANA, evades detection by repressing lytic viral replication to remain “below the radar” of adaptive immune defenses during host colonization.

## INTRODUCTION

The gammaherpesvirus (GHV) subfamily of herpesviruses is a large group of enveloped viruses that contain a double-stranded DNA genome. GHVs exhibit a biphasic infection cycle, which includes a lytic and a latent stage of viral infection (1). The lytic, or productive, phase is characterized by viral genome replication and generation of viral progeny. Lytic replication allows GHVs to traverse host mucosal barriers for dissemination and transmission in a proinflammatory manner, ultimately resulting in cell death and virion production (2). After lytic infection is cleared, GHVs establish latency, a phase of infection in which the viral genome is maintained in the host-cell nucleus as an extrachromosomal, covalently closed dsDNA episome, and the production of viral progeny is ceased. The transition to latency allows the virus to evade immune surveillance and persist for the lifetime of the host; however, specific stimuli can induce viral reactivation wherein the virus reinitiates lytic gene expression and restarts the productive replication cycle. Latency is likely minimally immunostimulatory, as viral transcription is limited to a few genes that promote infected cell survival and facilitate viral genome maintenance within host cells.

Kaposi sarcoma-associated herpesvirus (KSHV, also known as human herpesvirus 8, HHV-8) is a human GHV that establishes latent infection in B lymphocytes and other cell types and is linked to lymphoproliferative malignancies and cancer (3). For KSHV, seroprevalence varies based on geographical location; however, seropositivity ranges from 50% to 70% in endemic regions like sub-Saharan Africa and in people living with HIV infection (PLWH) (4). While GHV infections are not always linked to severe disease, KSHV-infected patients can develop primary effusion lymphoma, multicentric Castleman disease, the AIDS-defining endothelial cell malignancy, Kaposi sarcoma, and KSHV inflammatory cytokine syndrome (5).

How adaptive immune responses develop during primary KSHV infection of humans is virtually unknown, and this is likely because primary KSHV infection is asymptomatic with no signs of clinical disease. Studies conducted in KSHV endemic populations indicate that healthy individuals develop relatively weak immune responses to KSHV infection. Antibody responses in individuals with low or no detectable HIV viral loads and no previous history of KSHV-associated diseases are only weakly reactive to most of the KSHV proteome with few consistently recognized proteins (6). Furthermore, in KSHV endemic areas such as rural Uganda, the abundance of KSHV-specific CD4^+^ and CD8^+^ T cells is low in infected individuals and targets only a few immunodominant peptides (7). These findings suggest that KSHV is well controlled in healthy individuals and may have evolved to establish chronic infection in humans without eliciting potent adaptive immune responses. While several immunomodulatory proteins are encoded by KSHV, which of these viral gene products help KSHV avoid potently stimulating adaptive immunity is not known, especially *in vivo*.

Since chronic GHV infection can lead to cancer, it is critical that we understand the viral factors that promote latent infection and immune evasion. For KSHV, the latency-associated nuclear antigen (LANA) is encoded by the *ORF73* gene and is essential for latency establishment and viral episome persistence by tethering the viral genome to host chromatin (8, 9). In addition to maintaining the viral genome in infected cells, KSHV LANA (kLANA) suppresses lytic replication by repressing the lytic switch protein, replication and transcription activator (RTA), through modifications to viral chromatin during latency (10). Indeed, during *de novo* KSHV infection of SLK cells, kLANA recruits polycomb repressor complex 2 (PRC2) to the viral genome to silence lytic promoters, including the promoter for the RTA-encoding *ORF50* gene (11), suggesting that inhibiting lytic replication is an important event in primary KSHV infection and latency establishment.

Although GHVs exhibit a strict host restriction, several conserved proteins, including LANA, are functionally interchangeable between KSHV and murine gammaherpesvirus 68 (MHV68), a genetically related rodent virus frequently used to understand GHV pathogenesis *in vivo*. For instance, using an MHV68 recombinant in which the MHV68 LANA (mLANA)-encoding *ORF73* gene was replaced with the kLANA-encoding *ORF73* gene, we and others demonstrated that mLANA and kLANA are functionally interchangeable to enable latency establishment and long-term viral maintenance following MHV68 infection of mice (12, 13). Despite efficient latency establishment by the kLANA knock-in (KLKI) MHV68, we also observed that kLANA, but not mLANA, repressed the MHV68 *ORF50* promoter and limited RTA expression and subsequent lytic replication and reactivation (12). This finding revealed that the capacity of kLANA to suppress productive viral replication was transferable to MHV68. We also found that KLKI MHV68 did not cause splenomegaly, an infectious-mononucleosis (IM)-like pathology that is dependent on MHV68-driven CD4^+^ T cell stimulation and correlates with polyclonal lymphocyte activation (14).

In addition to promoting an IM-like syndrome, MHV68 generally elicits a potent virus-specific adaptive immune response that is characterized by widespread B and T cell activation targeting multiple viral antigens (15–17). In contrast to wild-type (WT) MHV68, replication-defective viruses like RTA-null (50.Stop) MHV68 minimally elicit adaptive immune activation and require multiple high-dose inoculations to promote virus-specific immunity (18, 19). Similarly, blockade of MHV68 replication with antiviral drugs also prevents virus-specific immunity (20). Together, these findings suggest the hypothesis that lytic GHV replication, and perhaps associated inflammation, is critical for stimulating virus-specific adaptive immunity. Given that kLANA suppresses lytic viral replication and immunity to primary KSHV infection is weak, we hypothesized that kLANA-mediated suppression of lytic replication is immunoevasive and serves to limit GHV-specific immune responses. By integrating MHV68 immunology with the KLKI MHV68 model, we sought to define how the amplitude of lytic replication, as dictated by kLANA, influences adaptive immune activation during MHV68 primary infection and latency establishment.

## RESULTS

### kLANA-mediated repression of *mORF50* restricts viral antigen accumulation during MHV68 lytic replication

**Fig 1:**
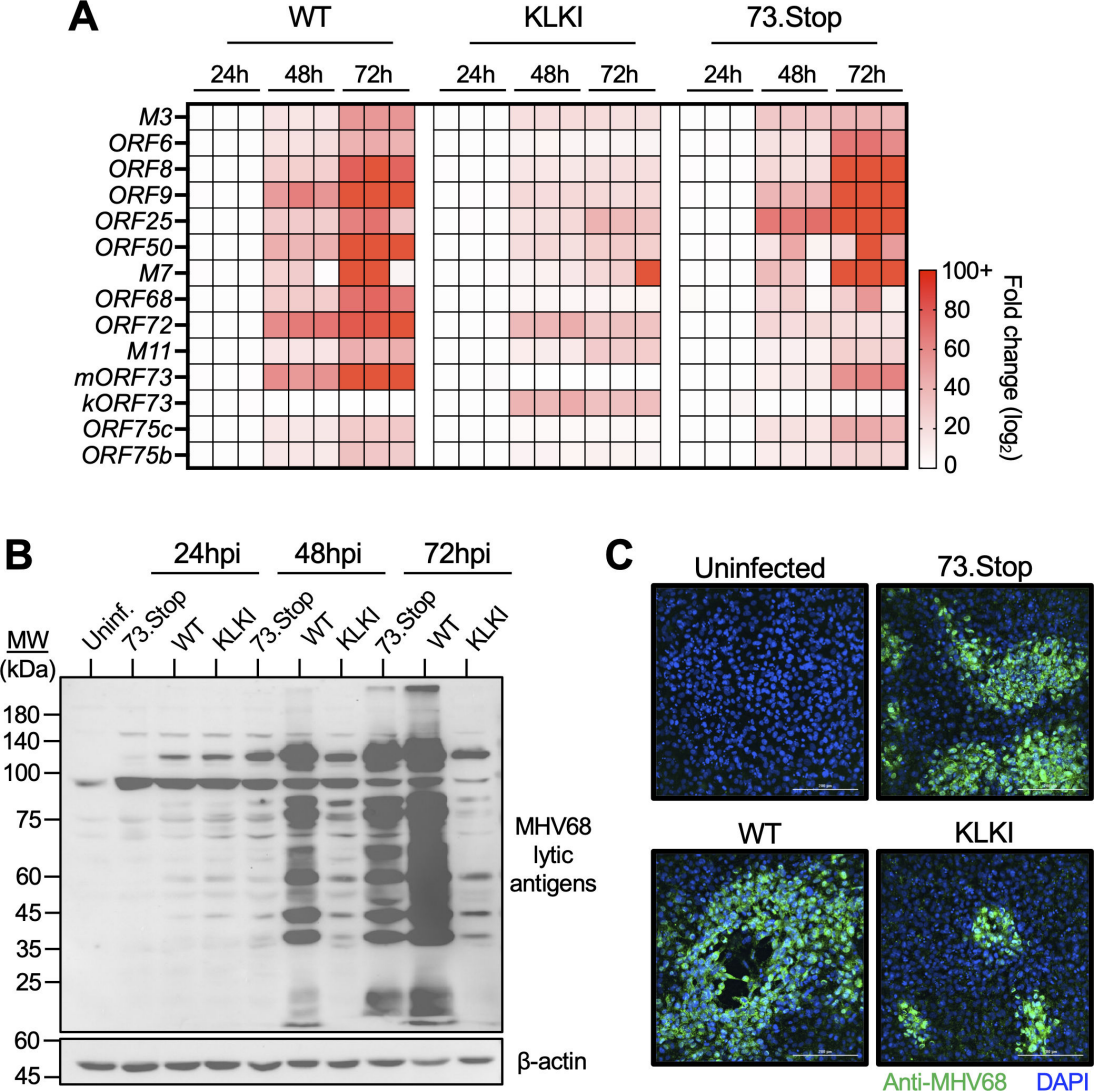
Expression of kLANA correlates with reduced lytic antigen accumulation during MHV68 infection. 3T12 fibroblasts were infected with the indicated viruses at an MOI of 0.1 PFU/cell. (**A**) RNA was collected at the indicated times postinfection. Viral transcripts were quantified by qRT-PCR relative to *B2m* using the ΔΔCT method and normalized to transcript abundance at 24 h postinfection. (**B**) Lysates were collected at the indicated times postinfection, and immunoblot analyses were performed using antibodies that recognize the indicated proteins. (**C**) Cells were fixed at 72 h postinfection and stained for indirect immunofluorescence analysis using antibodies that recognize the indicated proteins. DNA was stained with DAPI. Scale bar, 200 μm.

We previously demonstrated that kLANA, but not mLANA, has the capacity to repress MHV68 *ORF50* (mRTA) transcription. For a chimeric MHV68 virus in which mLANA was replaced with kLANA, this function correlated with suppressed MHV68 lytic replication, a phenotype that was overcome by providing mRTA in *trans* (12). To more broadly define how kLANA impacts MHV68 transcription, we performed quantitative RT-PCR (qRT-PCR) comparing WT, KLKI, and mLANA-deficient 73.Stop MHV68 transcription over time during lytic infection (21). Although LANA-null MHV68 is attenuated in murine fibroblasts following low-MOI infections, mLANA-null virus retains its capacity to progressively express viral gene products in a time-dependent manner (22, 23). Both WT and 73.Stop MHV68 exhibited the expected progression through the lytic transcription cascade, with viral transcripts increasing in abundance over time (Fig. 1A). In contrast, KLKI MHV68 infection was characterized by low-level lytic transcription that did not appear to amplify as infection progressed. Immunoblot analyses of infected cell lysates supported this observation, as lytic viral antigens detected with MHV68 immune sera were less abundant in cells infected with KLKI MHV68 compared to WT and 73.Stop viruses (Fig. 1B). Consistent with the notion that the amount of lytic viral gene expression is inherently linked to productive virus replication and cell-to-cell spread, KLKI MHV68 exhibited a small plaque phenotype relative to WT and mLANA-deficient viruses (Fig. 1C). In agreement with mRTA provided in *trans* rescuing KLKI MHV68 replication (12), infection of cells stably expressing mRTA equilibrated KLKI MHV68 transcription to levels approximating those of WT and 73.Stop MHV68 and enhanced the production of lytic viral antigens (Fig. S1A and B). These data indicate that expression of kLANA in place of mLANA correlates with suppressed viral transcription and decreased production of lytic antigens during productive MHV68 replication. In light of our prior work (12) and enhanced viral gene transcription and translation occurring in mRTA-expressing cells, this finding is consistent with kLANA-mediating suppression of *ORF50* transcription.

### KLKI MHV68 infection minimally stimulates B and T cell activation and immunity

Similar to IM after Epstein-Barr virus (EBV) infection of adolescent and adult humans, infection of mice with WT MHV68 causes splenomegaly, an immune-driven pathology reminiscent of IM that is characterized by immune cell recruitment and proliferation resulting in an increase in spleen mass. In contrast, KSHV is not known to cause splenomegaly during primary infection. We previously demonstrated that although KLKI MHV68 disseminated systemically and established latency in the spleen at levels equivalent to WT virus, KLKI MHV68 did not cause splenomegaly and only minimally reactivated from latency when compared to WT virus infections (12), phenotypes we confirmed in support of this study (see Fig. 4A and F and Fig. S2).

**Fig 2:**
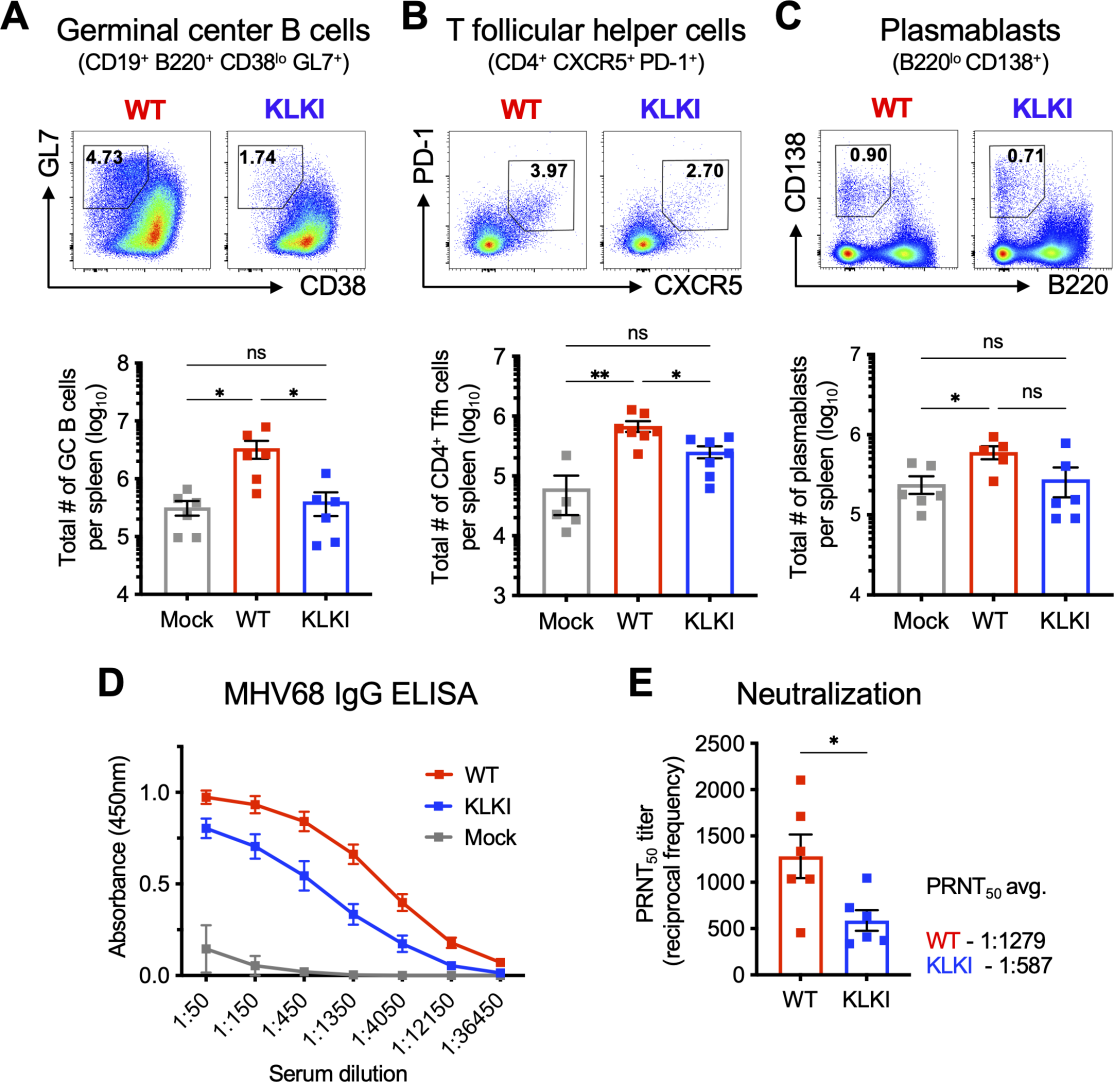
B cell activation and functional antibody responses are reduced following KLKI MHV68 infection. C57BL/6 mice were mock-infected or infected IN with 1,000 PFU of the indicated virus. Mice were sacrificed on days 16–18 postinfection. Spleens from infected mice were analyzed via flow cytometry. Representative gates from each infection are shown (top), and total cell counts were quantified (bottom). (**A**) Germinal center B cells were defined as CD19^+^/B220^+^/CD38^lo^/GL7^+^, (**B**) T follicular helper cells as CD3e^+^/CD4^+^/CXCR5^+^/PD-1^+^, and (**C**) plasmablasts as B220^lo^/CD138^+^. (**D and E**) Serum was isolated from infected animals for downstream analysis. (**D**) MHV68-specific IgG was determined by ELISA. (**E**) Neutralizing capacity of MHV68-specific antibodies was determined by plaque-reduction neutralization test (PRNT) assays using the indicated concentrations of the serum. PRNT_50_ titers were determined, where each sample neutralizes 50% of plaques on an indicator monolayer relative to negative controls. Groups of 3–5 mice were used for each infection and analysis. The results are means of 2–3 independent infections. (**A through C**) Each dot represents one mouse. Error bars represent the standard error of the mean. * and ** denote *P* < 0.05 and *P* < 0.01, respectively, in a one-way ANOVA with Tukey’s test. ns, not significant. (**E**) * denotes *P* < 0.05 in an unpaired Student’s *t*-test.

Since splenomegaly is an immune-mediated pathology and cells infected with KLKI MHV68 generate fewer lytic viral antigens over time, we hypothesized that the presence of kLANA and the related suppression of lytic viral gene expression would reduce immune stimulation by KLKI MHV68 relative to the WT virus. To test this hypothesis, we compared B and T cell populations in the spleen on days 16–18 after intranasal infection with 1,000 PFU of either WT or KLKI MHV68. This dose of virus and time point typically correlate with potent immune activation after WT MHV68 infection. Consistent with previous reports (14, 24, 25), infection with WT MHV68 elicited a ~2-fold increase in cellularity in the spleen that correlated with modest, but not significant, increases in total B cells (CD19^+^ B220^+^), T cells (CD3^+^), and CD4^+^ or CD8^+^ T cells (CD3^+^ CD4^+^ or CD8^+^), whereas KLKI MHV68 infections most closely resembled naive spleens (Fig. S3). When germinal center (GC) B cells (CD19^+^ B220^+^ GL7^+^ CD38^lo^) and T follicular helper cells (Tfh, CD4^+^ CXCR5^+^ PD-1^+^) were analyzed, expansion of both cell types was potently stimulated by the WT virus, whereas neither cell type was significantly increased relative to naive mice following KLKI MHV68 infection (Fig. 2A and B). Plasmablast (CD138^+^ B220^lo^) expansion was also lower following KLKI MHV68 infection; however, the reduction was not significant compared to the WT virus (Fig. 2C). Together, these data suggest that KLKI MHV68 infection establishes latency without triggering potent B cell activation and GC B cell expansion.

As part of the adaptive immune response, GC B cell and plasmablast expansion typically leads to the production of virus-specific antibodies that help control viral infection. While specific targets are incompletely defined, antibodies against MHV68 proteins correlate with vaccine-mediated protection, and passive serum transfer protects B cell-deficient mice from lethal MHV68 disease (26, 27). To assess the antiviral impact of reduced B cell activation during KLKI MHV68 infection, we compared MHV68-specific humoral responses after WT and KLKI MHV68 infections. We quantified MHV68-specific IgG in mouse sera by ELISA, finding that virus-specific antibodies generated in response to KLKI MHV68 infection had a reduced capacity to recognize MHV68 lytic antigens (Fig. 2D). To assess antibody functionality, PRNT assays were conducted to determine the neutralizing capacity of virus-specific Ig. PRNT_50_ titers were 1:587 for KLKI-infected animals, while WT MHV68 infection resulted in a PRNT_50_ titer of 1:1279, indicating that the humoral response to KLKI infection was ~2.2-fold less potent than the response to WT MHV68 infection (Fig. 2E). Collectively, these data indicate that B cell activation and humoral immunity are less potent during latency establishment by KLKI MHV68 relative to the WT virus.

**Fig 3:**
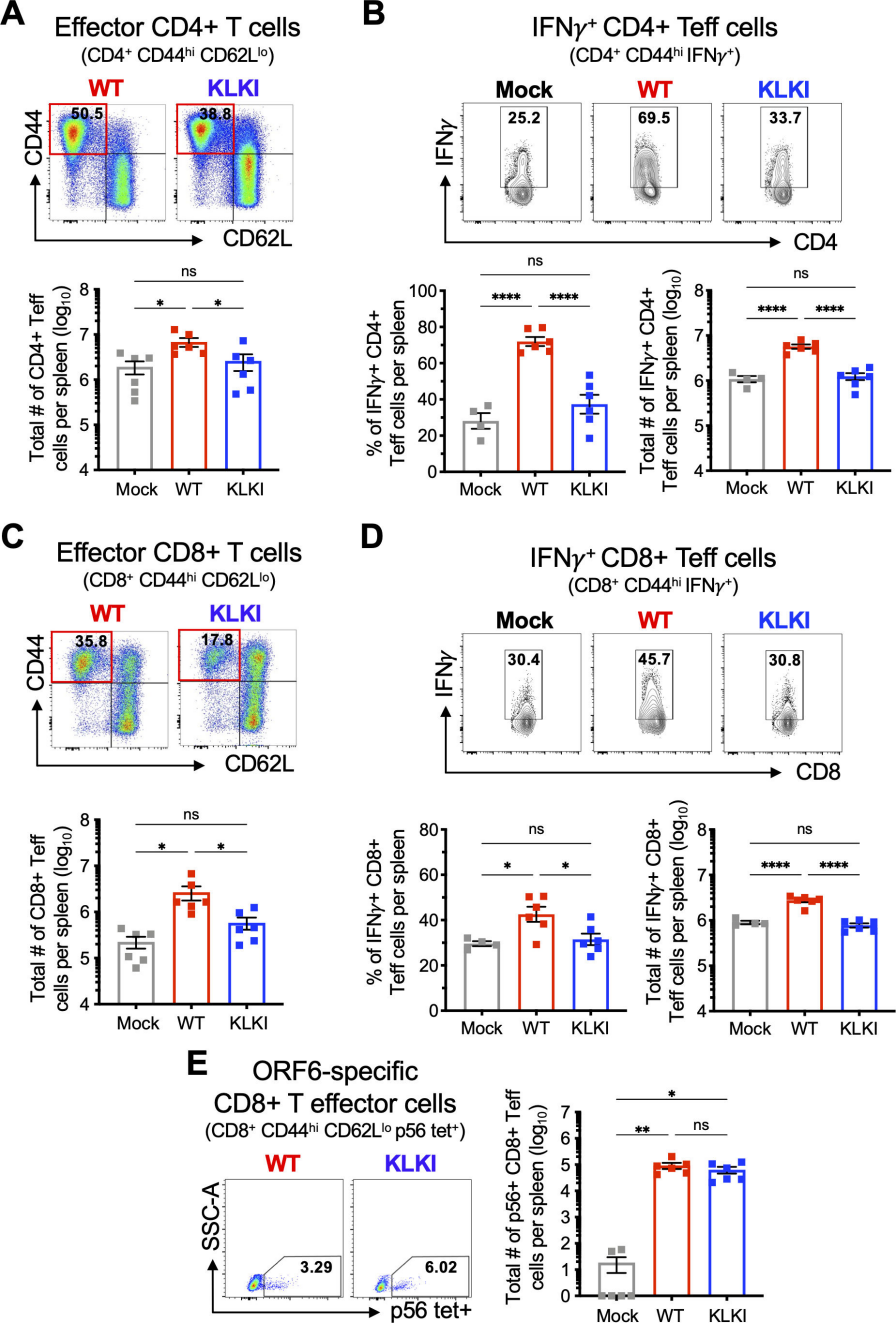
Virus-specific CD8^+^ T cell responses arise during KLKI MHV68 infection despite limited T cell activation and IFN-γ production. C57BL/6 mice were mock-infected or infected IN with 1,000 PFU of the indicated virus. Mice were sacrificed on days 16–18 postinfection. Spleens from infected mice were analyzed via flow cytometry. For cytokine restimulation experiments in B and D, spleen cells were restimulated *ex vivo* with PMA/ionomycin for 5 h prior to analysis. Representative gates from each infection are shown (top), and total cell counts and frequencies were quantified (bottom). (**A**) Effector CD4+ T cells were gated as CD3e^+^/CD4^+^/CD44^hi^/CD62L^lo^, (**B**) IFN-γ-producing effector CD4+ T cells as CD3e^+^/CD4^+^/CD44^hi^/IFNγ^+^, (**C**) effector CD8^+^ T cells as CD3e^+^/CD8^+^/CD44^hi^/CD62L^lo^, (**D**) IFNγ-producing effector CD8^+^ T cells as CD3e^+^/CD8^+^/CD44^hi^/IFNγ^+^, and (**E**) ORF6-specific effector CD8^+^ T cells as CD3e^+^/CD8^+^/CD44^hi^/CD62L^lo^/p56 tetramer^+^. Groups of 2–5 mice were used for each infection and analysis. Each dot represents one mouse. The results are means of 2 or 3 independent infections. Error bars represent the standard error of the mean. * and ** denote *P* < 0.05 and *P* < 0.01, respectively, in a one-way ANOVA with Tukey’s test. ns, not significant. Fluorescence minus one (FMO) controls are shown in Fig. S4.

Effector CD4^+^ and CD8^+^ T cell expansion in the spleen contributes to splenic inflammation; CD4^+^ T cells that produce IFN-γ play a vital role in controlling MHV68 replication during chronic infection (28–30), and CD8^+^ T cells directly recognize and kill infected cells through perforin and granzyme production, while also secreting TNF-α and IFN-γ to drive antiviral inflammatory responses (31, 32). In flow cytometry-based comparisons of T cell activation following KLKI and WT MHV68 infection, effector CD4^+^ and CD8^+^ T cell compartments (CD4^+^/CD8^+^ CD44^hi^ CD62L^lo^) were much smaller in KLKI MHV68-infected animals (Fig. 3A and C). This correlated with significantly different frequencies of IFNγ producing effector populations for both CD4^+^ and CD8^+^ T cells (Fig. 3B and D), suggesting that antiviral control mediated by these cells is not engaged following KLKI MHV68 infections. However, similar levels of an immunodominant effector CD8^+^ T cell population that recognizes the p56 MHC class I tetramer containing the ORF6_487-495_ peptide were induced by both viruses (Fig. 3E). Thus, despite reduced lytic replication and reactivation (Fig. S2; 12) and minimal lymphocyte activation in general, KLKI MHV68 drives virus-specific T cell responses to an immunodominant MHV68 T cell epitope, which is consistent with data from replication-defective viruses as well (18, 19). Overall, these data suggest that repression of lytic replication by kLANA during KLKI MHV68 infection correlates with diminished effector T cell responses, whereas a virus-specific immunodominant CD8^+^ T cell response still occurs.

### Inoculating dose is not a major determinant of adaptive immune activation

While the infectious dose for KSHV in humans is not known, a dose-response analysis of MHV68 infection after intranasal infection found that inoculation with doses ranging from 0.1 to 400,000 PFU results in equivalent levels of latent infection in the spleens of mice (33). However, the impact that the initial inoculating dose of any GHV has on immune activation is not defined. We reasoned that reduced lytic replication and reactivation by KLKI MHV68 could reduce the amount of danger signaling and antigen availability for potent immune activation, leading us to hypothesize that a high-dose inoculation of KLKI MHV68 infection would enhance immune activation and potency. We therefore performed a dose-response analysis for WT and KLKI MHV68 using infectious doses of 10, 1,000, and 100,000 PFU and assessed adaptive immune responses.

**Fig 4:**
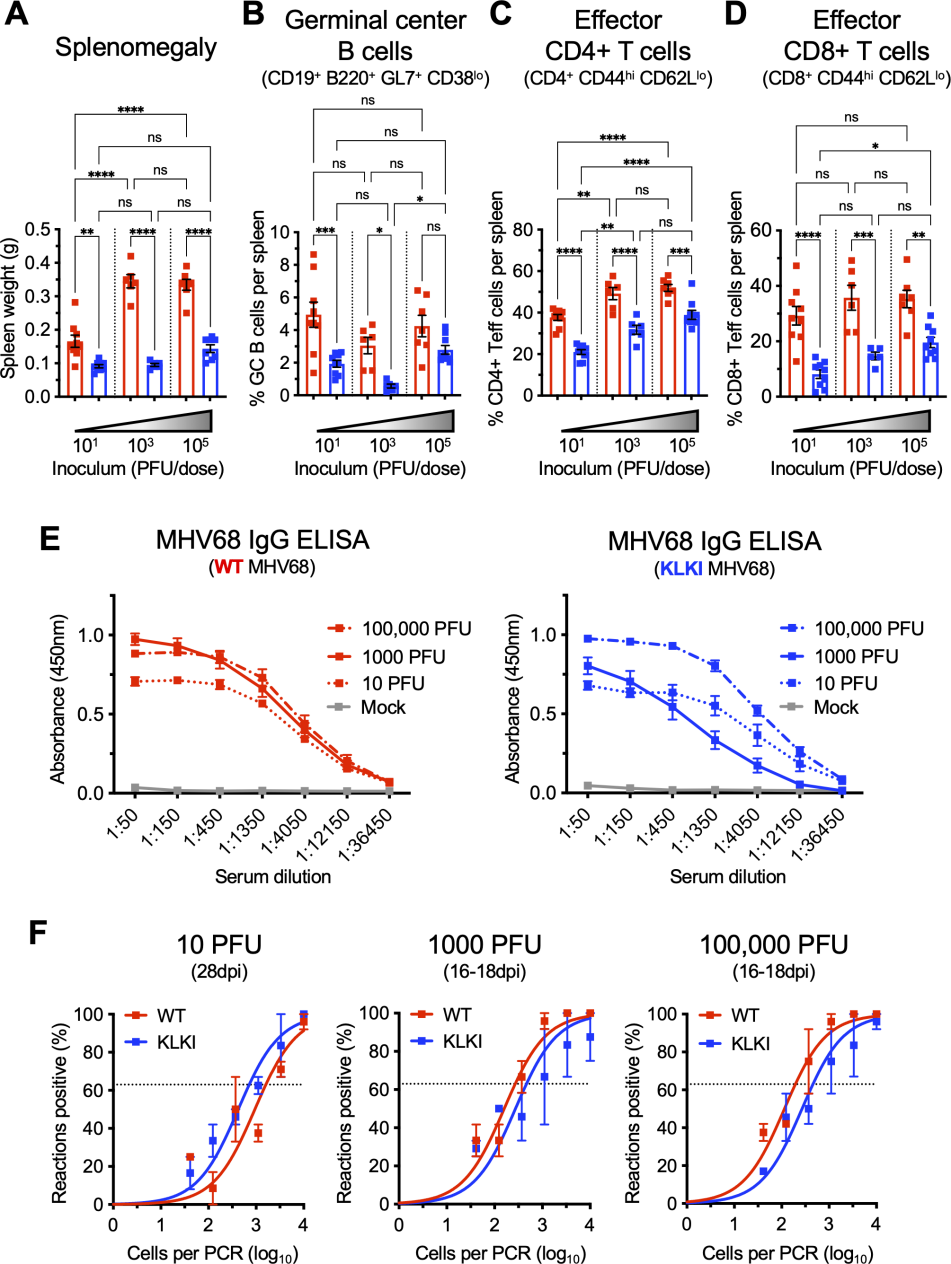
Adaptive immune activation in response to KLKI MHV68 infection is reduced regardless of the infectious dose. C57BL/6 mice were infected IN with 10, 1,000, or 100,000 PFU of the indicated virus. Mice were sacrificed on days 16–18 postinfection (1,000 or 100,000 PFU) or day 28 postinfection (10 PFU). (**A**) Spleens were weighed as an assessment of splenomegaly at each dose tested, and the percentages of (**B**) germinal center B cells (CD19^+^/B220^+^/CD38^lo^/GL7^+^), (**C**) effector CD4^+^ T cells (CD3e^+^/CD4^+^/CD44^hi^/CD62L^lo^), and (**D**) effector CD8^+^ T cells (CD3e^+^/CD8^+^/CD44^hi^/CD62L^lo^) from infected spleens were analyzed and quantified via flow cytometry. (**E**) Serum was isolated from infected animals and analyzed in MHV68-specific IgG ELISAs for WT MHV68 (red; left) or KLKI MHV68 (blue; right) infections at each dose. (**F**) Single-cell suspensions of splenocytes were serially diluted, and the frequencies of cells harboring MHV68 genomes were determined using a limiting-dilution PCR analysis. Reactivation frequencies are shown in Fig. S2. Groups of 3–5 mice were used for each infection and analysis. The results are means of 2 or 3 independent infections. (**A through D**) Each dot represents one mouse. Error bars represent the standard error of the mean. *, **, ***, and **** denote *P* < 0.05, *P* < 0.01, *P* < 0.001, and *P* < 0.0001, respectively, in a one-way ANOVA with Tukey’s test. ns, not significant.

Splenomegaly induced by KLKI MHV68 remained minimal, even following inoculation with 100,000 PFU, and enumeration of immune cell subsets in spleens indicated that the percentages of GC B cells, effector CD4^+^, and effector CD8^+^ T cells were significantly lower during KLKI MHV68 infection compared to WT virus infections for all doses tested (Fig. 4A through D). Effector CD4^+^ and CD8^+^ T cell percentages only modestly increased in a dose-dependent manner for KLKI MHV68 infections, while WT virus infections elicited high levels of effector T cell subsets independently of the inoculating dose (Fig. 4C and D). While WT MHV68 infection stimulated a more potent IgG antibody response at both low and intermediate doses, virus-specific antibody responses were comparable between WT and KLKI MHV68 infection only for the 100,000 PFU dose (Fig. 4E). Importantly, the frequencies of latently infected cells in spleens were comparable between both viruses at all doses tested (Fig. 4F; Table 1). Note that evaluations for 10 PFU infections required analysis on day 28 post-infection, as the virus was not present in spleens at the traditional day 16–18 time point (not shown). Together, these data demonstrate that although virus-specific antibody production increased in a dose-dependent manner for KLKI MHV68, general immune activation was predominantly independent of inoculating dose.

**TABLE 1:** Frequencies of cells harboring MHV68 genomes in latently infected mice

Virus	Mouse strain	Dose (PFU)	Harvest day post-infection	No. of MHV68 genome-positive cells/total no. of cells
WT MHV68	WT C57BL/6	10	28	1/1,600
WT MHV68	WT C57BL/6	1,000	16–18	1/270
WT MHV68	WT C57BL/6	100,000	16–18	1/200
KLKI MHV68	WT C57BL/6	10	28	1/470
KLKI MHV68	WT C57BL/6	1,000	16–18	1/730
KLKI MHV68	WT C57BL/6	100,000	16–18	1/750
WT MHV68	Ifnar1^−/−^	1,000	16–17	1/66
KLKI MHV68	Ifnar1^−/−^	1,000	16–17	1/67

### Abrogation of type I IFN signaling enhances productive viral replication during KLKI MHV68 infection

Since high-dose KLKI MHV68 infection did not promote activation of the adaptive immune response to the same extent as WT MHV68 infection, we reasoned that a lytic replication threshold and its corresponding accumulation of viral antigens need to be breached to potently activate adaptive immune responses during MHV68 infection. We therefore sought to enhance KLKI lytic replication to evaluate the impact on adaptive immune stimulation. MHV68 lytic replication is controlled by type I IFN responses (34, 35). Thus, we hypothesized that KLKI MHV68 lytic replication would be enhanced in type I IFN non-responsive cells in a manner that surpasses the immune engagement threshold.

**Fig 5:**
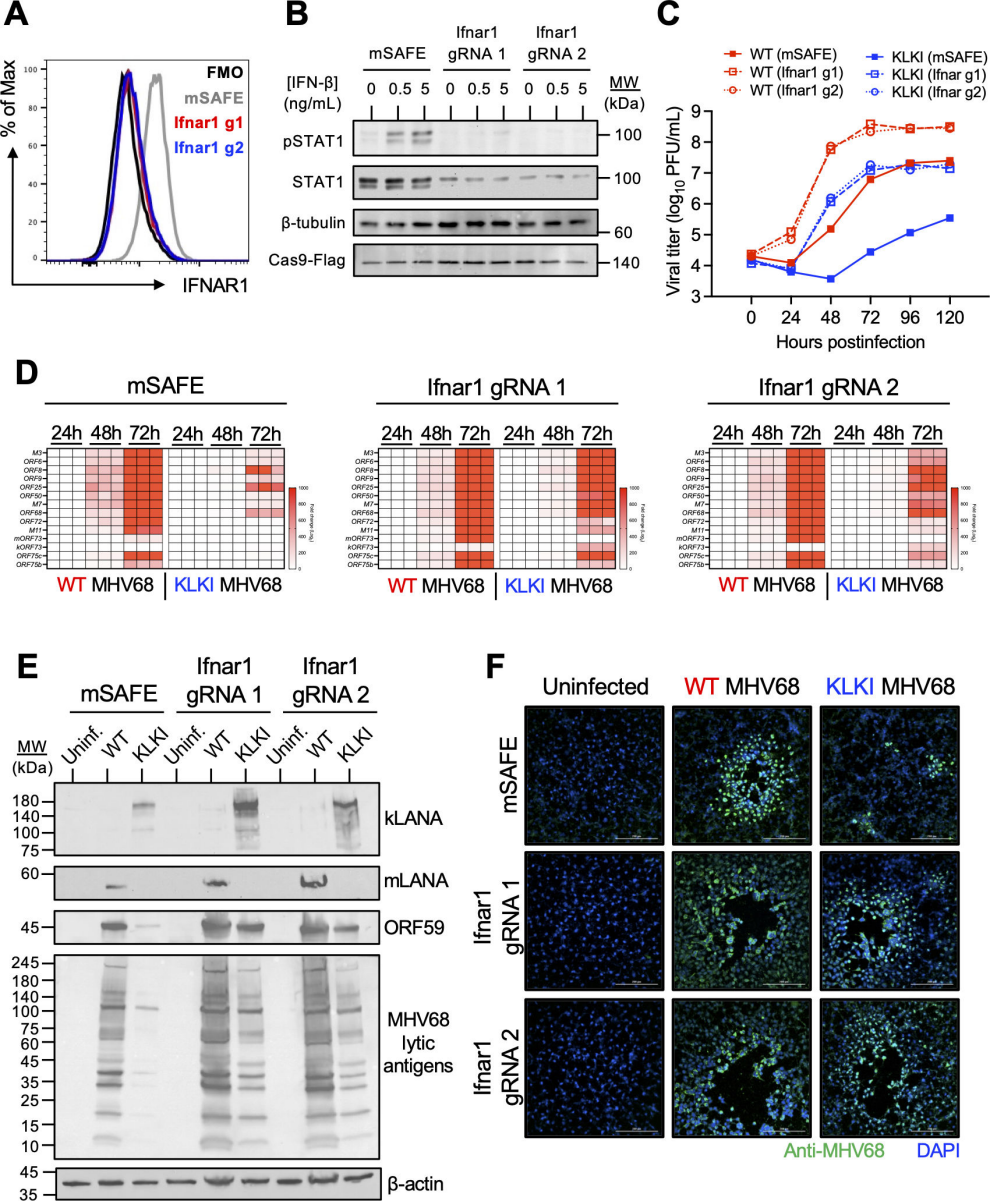
*Ifnar1* ablation in murine fibroblasts promotes lytic antigen accumulation and viral replication during KLKI MHV68 infection. (**A**) *Ifnar1*-deficient or non-target control (mSAFE) 3T12 fibroblast pools were stained with *Ifnar1*-specific antibodies and analyzed by flow cytometry. (**B**) Cells were treated with recombinant IFN-β at the indicated doses to determine the efficacy of *Ifnar1*-specific deletion. Lysates were collected 2.5 h after treatment, and immunoblot analyses were performed using antibodies to detect the indicated proteins. (**C, D, and F**) Cells were infected with the indicated virus at an MOI of 0.1 PFU/cell. (**C**) Viral titers were determined at the indicated times postinfection by plaque assay. (**D**) RNA was collected at the indicated times postinfection. Viral transcripts were quantified by qRT-PCR relative to *B2m* using the ΔΔCT method and normalized to transcript abundance at 24 h postinfection. (**E**) Cells were infected at an MOI of 1 PFU/cell, and lysates were collected at 48 h postinfection. Immunoblot analyses were performed using antibodies that recognize the indicated proteins. (**F**) Cells were fixed 72 h postinfection and stained for indirect immunofluorescence analysis using antibodies that recognize the indicated proteins. DNA was stained with DAPI. Scale bar, 200 μm.

As an initial test, we utilized CRISPR-Cas9 gene editing to ablate the alpha subunit of the type I IFN receptor (*Ifnar1*) in 3T12 fibroblasts, rendering them type I IFN nonresponsive, and evaluated KLKI MHV68 infection relative to the WT virus (Fig. 5). *Ifnar1* ablation was confirmed by flow cytometry (Fig. 5A) and the lack of STAT1 phosphorylation (pSTAT1) after treatment with recombinant IFN-β (Fig. 5B). We then infected *Ifnar1*-deficient fibroblasts with either WT or KLKI MHV68 and evaluated viral transcription over time. While control fibroblasts infected with KLKI MHV68 exhibited low-level lytic transcription that did not appear to amplify to the same extent as WT virus infections, both viruses readily progressed through the lytic cascade following infection of *Ifnar1*-deficient cells (Fig. 5D). Immunoblot and immunofluorescence analyses revealed that lytic viral gene products and plaque size were dramatically increased, respectively, following KLKI MHV68 infection of *Ifnar1*-deficient cells relative to mSAFE non-target control cells (Fig. 5E and F). Consistent with the observation that lytic viral gene transcription and translation were enhanced in the absence of type I IFN signaling, WT and KLKI MHV68 replicated more efficiently in *Ifnar1*-deficient fibroblasts (Fig. 5C). While still reduced compared to WT MHV68, a phenotype we attribute to continued kLANA-mediated suppression of *ORF50*/RTA despite IFN non-responsiveness, these results demonstrate that *Ifnar1* deletion potently enhances viral antigen accumulation and viral replication during KLKI MHV68 infection.

**Fig 6:**
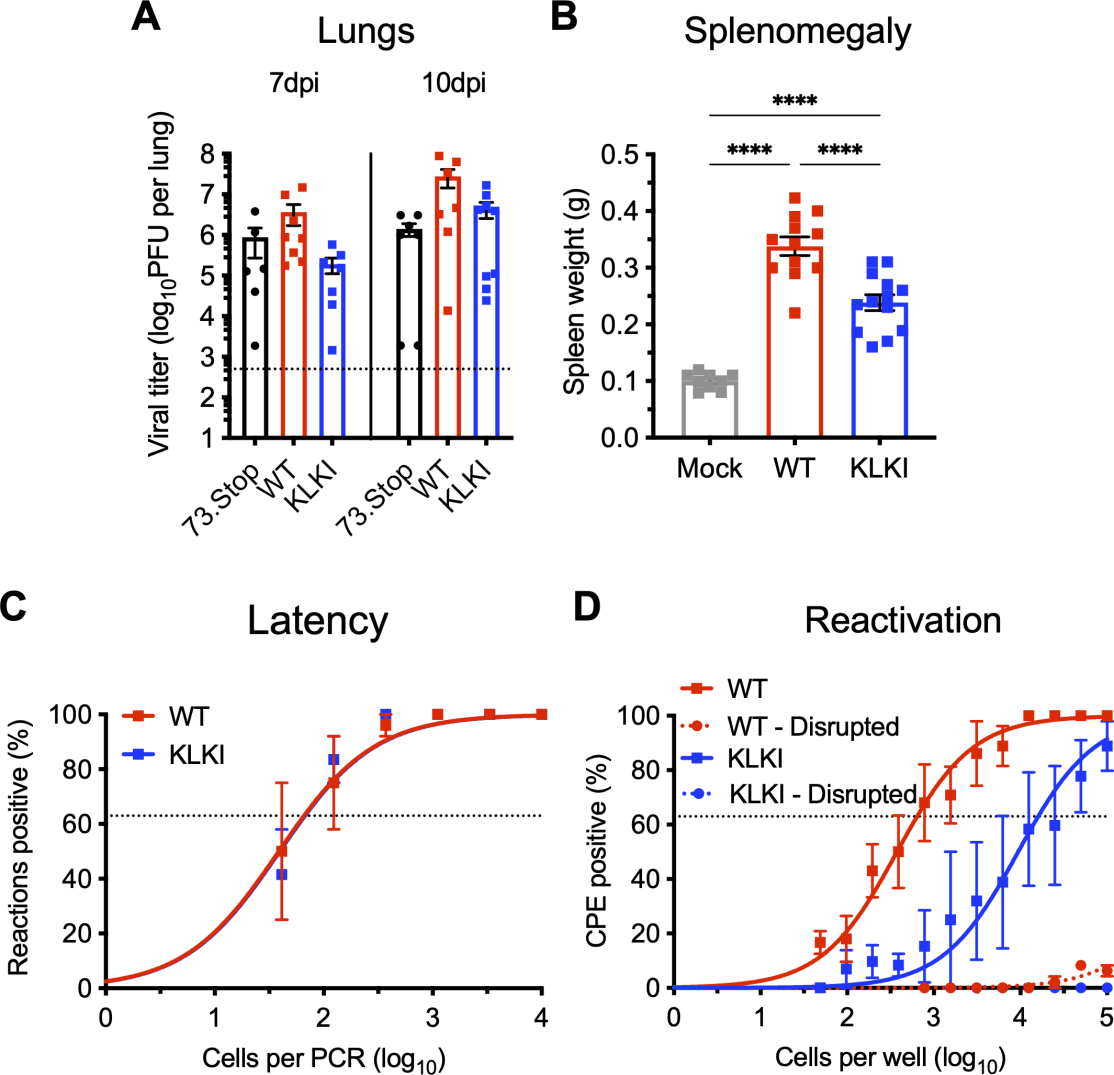
Lytic replication is enhanced during KLKI MHV68 infection of *Ifnar1^−/−^* mice and promotes splenomegaly. *Ifnar1^−/−^* mice were mock-infected or infected IN with 1,000 PFU of the indicated virus. (**A**) Mice were sacrificed on days 7 or 10 postinfection. Lungs were homogenized, and viral titers were determined by plaque assay. (**B through D**) Mice were sacrificed on days 16–17 postinfection. (**B**) Spleens were weighed as an assessment of splenomegaly. (**C**) Single-cell suspensions of splenocytes were serially diluted, and the frequencies of cells harboring MHV68 genomes were determined using a limiting-dilution PCR analysis. (**D**) Reactivation frequencies were determined by *ex vivo* plating of serially diluted splenocytes on an indicator monolayer. Cytopathic effect was scored 1 week postplating. Groups of 3–5 mice were infected, and splenocytes were pooled for analysis (**C and D**). The results are means of 2 or 3 independent infections. Error bars represent the standard error of the mean. *** and **** denote *P* < 0.001 and *P* < 0.0001, respectively, in a one-way ANOVA with Tukey’s test. ns, not significant.

To test the immune engagement threshold hypothesis *in vivo*, we infected *Ifnar1^−/−^* C57BL/6 mice with WT and KLKI MHV68 and evaluated viral replication, latency, and adaptive immunity. Consistent with previous studies (36), after IN inoculation WT MHV68 viral titers in lungs were approximately 100-fold higher in *Ifnar1*^−/−^ mice than WT C57BL/6 mice at day 7 post-infection and exhibited delayed clearance on day 10 post-infection (Fig. 6A). This was also the case for KLKI MHV68. Although still reduced compared to WT virus, splenomegaly did occur following KLKI MHV68 infection of *Ifnar1*^−/−^ mice with spleen weights significantly higher than uninfected controls (Fig. 6B). As in WT C57BL/6 mice (Fig. 4E), both KLKI and WT MHV68 established latency equivalently in spleens of *Ifnar1*^−/−^ mice on days 16–17 post-infection (Fig. 6C and Table 1). While KLKI MHV68 reactivation from latency remained reduced compared to WT virus, both WT and KLKI MHV68 exhibited an increased frequency of reactivating cells in comparison to WT C57BL/6 mice, and no persistent viral replication was observed (Fig. 6D, Table 2, and Fig. S2). These data indicate that the absence of type I IFN signaling in *Ifnar1^−/−^* mice enhances KLKI MHV68 lytic replication and reactivation and restores splenomegaly.

**TABLE 2:** Frequencies of latently infected cells reactivating *ex vivo*^*a*^

Virus	Mouse strain	Dose (PFU)	Harvest day	No. of reactivating cells/total no. of cells
WT MHV68	WT C57BL/6	10	28	ND
WT MHV68	WT C57BL/6	1,000	16–18	1/8,700
WT MHV68	WT C57BL/6	100,000	16–18	1/8,300
KLKI MHV68	WT C57BL/6	10	28	ND
KLKI MHV68	WT C57BL/6	1,000	16–18	ND
KLKI MHV68	WT C57BL/6	100,000	16–18	ND
WT MHV68	Ifnar1^−/−^	1,000	16–17	1/650
KLKI MHV68	Ifnar1^−/−^	1,000	16–17	1/16,000

^*a*^ N.D., not determined (the frequency was below the limit of detection for the assay).

### KLKI MHV68 potently activates the adaptive immune response in *Ifnar1^−/−^* mice

**Fig 7:**
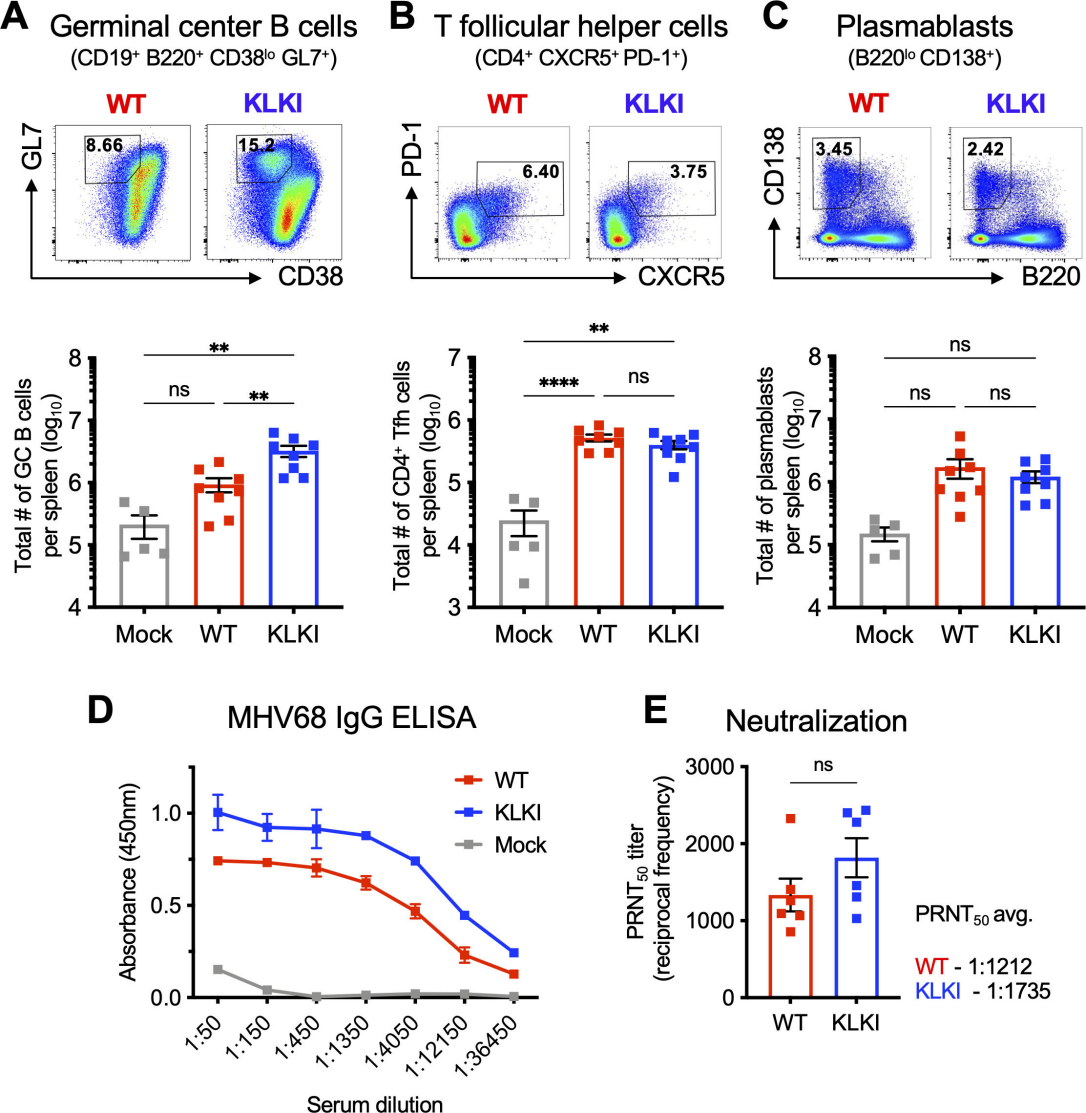
B cell activation and antibody responses are enhanced during KLKI MHV68 infection of *Ifnar1^−/−^* mice. *Ifnar1^−/−^* mice were infected IN with 1,000 PFU of the indicated virus. Mice were sacrificed on days 16–17 postinfection. Spleens from infected mice were analyzed via flow cytometry. Representative gates from each infection are shown (top), and total cell counts were quantified (bottom). (**A**) Germinal center B cells were defined as CD19^+^/B220^+^/CD38^lo^/GL7^+^, (**B**) T follicular helper cells as CD3e^+^/CD4^+^/CXCR5^+^/PD-1^+^, and (**C**) plasmablasts as B220^lo^/CD138^+^. (**D and E**) Serum was isolated from infected animals for downstream analysis. (**D**) MHV68-specific IgG was quantified by ELISA. (**E**) Neutralizing capacity of MHV68-specific antibodies was determined by PRNT assays using the indicated concentrations of serum. PRNT_50_ titers were determined, where each sample neutralizes 50% of plaques on an indicator monolayer relative to negative controls. Groups of 3–5 mice were used for each infection and analysis. The results are means of 2–3 independent infections. (**A through C**) Each dot represents one mouse. Error bars represent the standard error of the mean. ** and **** denote *P* < 0.01 and *P* < 0.0001, respectively, in a one-way ANOVA with Tukey’s test. ns, not significant.

Since MHV68-induced splenomegaly is dependent on T cell activation (14, 37), enhanced splenomegaly following infection of *Ifnar1*^−/−^ mice with KLKI MHV68 suggested that adaptive immune activation occurred in this model. Indeed, in contrast to findings in WT C57BL/6 mice, KLKI MHV68 infection caused potent GC B cell expansion in *Ifnar1*^−/−^ mice (Fig. 7A) that correlated with increased Tfh cell expansion when compared to mock-infected spleens (Fig. 7B). Additionally, both WT and KLKI MHV68 infections caused a 10-fold increase in plasmablast expansion relative to uninfected controls (Fig. 7C). In agreement with the B cell activation phenotypes, serum collected from both WT and KLKI MHV68-infected *Ifnar1^−/−^* mice contained high levels of MHV68-specific IgG, and both sera exhibited comparable capacities to neutralize MHV68 in PRNT_50_ assays (Fig. 7D and E).

**Fig 8:**
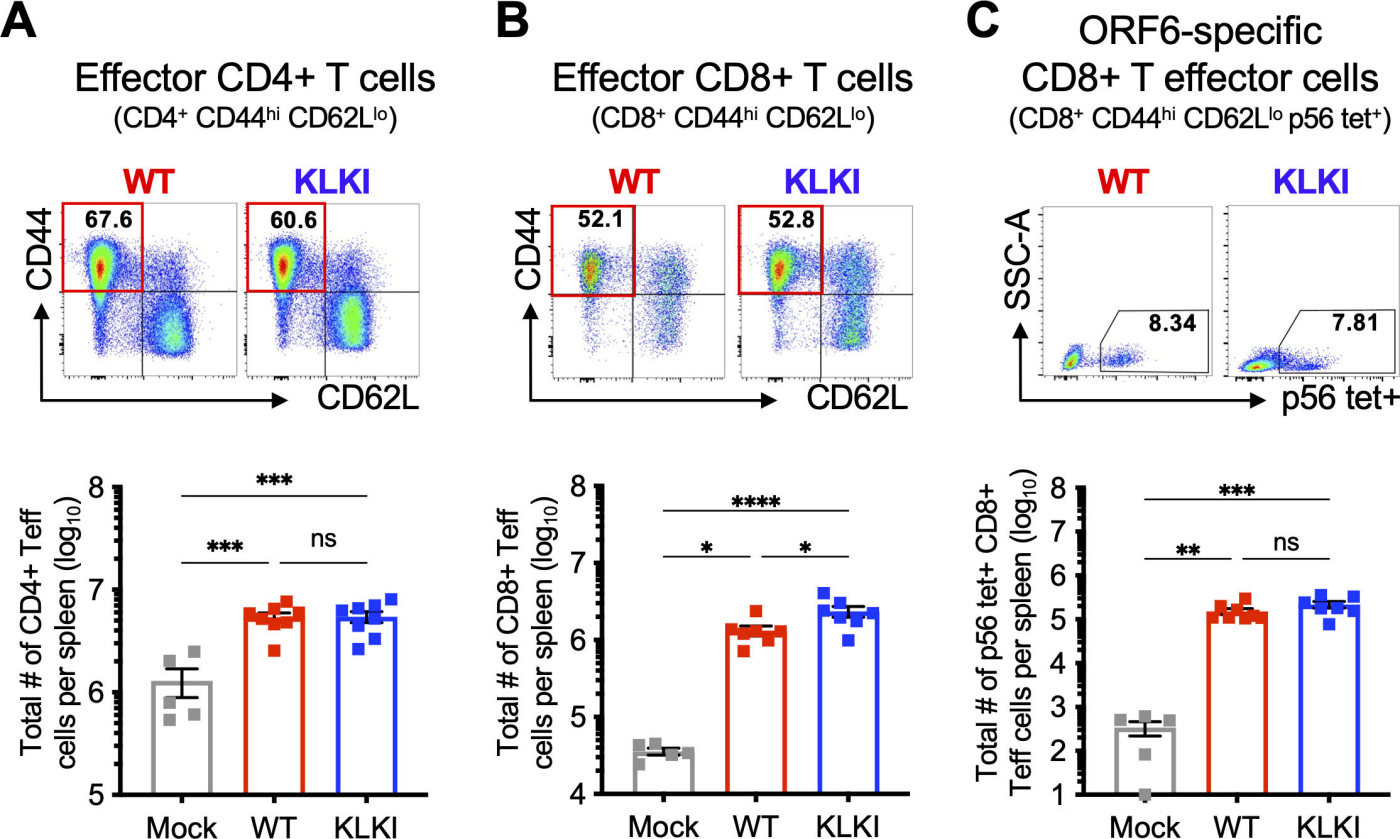
T cell activation and virus-specific effector CD8^+^ T cell responses are comparable following KLKI MHV68 infection of *Ifnar1^−/−^* mice. *Ifnar1^−/−^* mice were mock-infected or infected IN with 1,000 PFU of the indicated virus. Mice were sacrificed on days 16–17 postinfection. Spleens from infected mice were analyzed via flow cytometry. Representative gates from each infection are shown (top), and total cell counts were quantified (bottom). (**A**) Effector CD4^+^ T cells were gated as CD3e^+^/CD4^+^/CD44^hi^/CD62L^lo^, (**B**) effector CD8+ T cells as CD3e^+^/CD8^+^/CD44^hi^/CD62L^lo^, and (**C**) ORF6-specific effector CD8^+^ T cells as CD3e^+^/CD8^+^/CD44^hi^ /CD62L^lo^/p56 tetramer^+^. Groups of three to five mice were used for each infection and analysis. Each dot represents one mouse. The results are means of 2 or 3 independent infections. Error bars represent the standard error of the mean. *, **, ***, and **** denote *P* < 0.05, *P* < 0.01, *P* < 0.001, and *P* < 0.0001, respectively, in a one-way ANOVA with Tukey’s test. ns, not significant. Fluorescence minus one (FMO) controls are shown in Fig. S4.

Consistent with splenomegaly occurring following KLKI MHV68 infection of *Ifnar1^−/−^* mice, effector CD4^+^ T cell activation in spleens was similar for both WT and KLKI MHV68 (Fig. 8A), and effector CD8^+^ T cell populations also were similarly elevated following infection with either virus (Fig. 8B). Equivalent numbers of immunodominant p56 tetramer-specific effector CD8^+^ T cells were again elicited (Fig. 8C). Thus, KLKI MHV68 infection of *Ifnar1^−/−^* mice promotes B and T cell responses that are comparable to WT MHV68 infection. Taken together, these results suggest that the level of lytic replication/reactivation during MHV68 infection is a primary driver of adaptive immune engagement. Moreover, these findings provide evidence that suppression of lytic viral transcription and replication by kLANA functions in immune evasion, enabling efficient latency establishment without potent adaptive immune stimulation.

## DISCUSSION

Lytic replication facilitates GHV dissemination and transmission to new hosts; however, this process is highly immunogenic, promoting the detection and neutralization of infected cells by host immune responses. By switching to latency, GHVs minimize viral gene expression to factors that facilitate viral episome persistence and host cell survival, thereby evading host immunity. Here, we demonstrate that an MHV68 virus expressing kLANA in place of mLANA exhibits much reduced viral gene expression relative to WT and mLANA-deficient MHV68, suggestive of a pro-latent phenotype. Despite establishing latency at frequencies equivalent to WT MHV68, polyclonal and virus-specific adaptive immune responses were comparatively much lower following KLKI MHV68 infection of mice. Indeed, reductions in B and T cell activation during KLKI virus infections correlated with a reduction in splenomegaly, suggesting that proinflammatory responses during latency establishment are reduced when lytic replication and/or reactivation is suppressed. This interpretation is supported by IFNγ-producing T cells not being potently stimulated by KLKI MHV68. In general, type I IFN responses act as host restriction factors to viral replication, and deletion of the *Ifnar1* receptor enhances lytic MHV68 replication/reactivation. Consistent with this, infection of *Ifnar1^−/−^* mice resulted in enhanced KLKI MHV68 lytic replication and reactivation, infection-induced splenomegaly, and adaptive immune cell activation *in vivo*. Since enhanced lytic replication/reactivation during KLKI MHV68 infections coincided with stronger immune cell activation in *Ifnar1^−/−^* mice, we propose that a lytic replication threshold must be surpassed for potent adaptive immune cell activation in response to GHV infection.

It is important to note that we currently include reactivation within lymphoid organs in our overarching interpretation, as it is challenging to separate the immune-activating impacts of reactivation *in situ* from primary acute replication. The rationale for this interpretation is primarily based on the observation that KLKI MHV68 exhibits increased reactivation efficiency following infection of *Ifnar1^−/−^* mice. We attempted to address the contribution of reactivation more directly through conditional deletion of mRTA-encoding *ORF50 in vivo* as previously described (38). Although we previously reported reduced splenomegaly and reactivation relative to WT virus (38), impacts on immune activation following infection of CD19-Cre mice with floxed *ORF50* MHV68 were variable and inconclusive and therefore did not provide clarity (not shown). The converse argument that reactivation does not influence immune activation could also be made, given that following infection with 10 PFU of either WT or KLKI MHV68, reactivation remained below the limit of detection for *both* viruses; however, differences in adaptive immune activation were still observed. This seems to suggest that additional factors beyond measurable reactivation may drive these phenotypes. Suffice it to say, whether reactivation contributes directly to the phenotypes observed here remains unresolved.

LANA homologs mediate viral episome maintenance and segregation within latently infected cells. In addition to its essential functions in maintaining latent viral genomes, kLANA also represses *ORF50*/RTA transcription and subsequent lytic replication soon after the naked viral DNA is deposited in host cells upon *de novo* infection (39). Indeed, kLANA recruits repressive chromatin modifying enzymes to the KSHV genome, further suppressing viral transcription, as well as regulating host cell transcription (11, 40–43). Coupled with the facts that lytic gene expression is transient and tightly regulated during *de novo* KSHV infection (11, 39) and that KSHV primarily remains latent in a variety of cell types in culture (44), we hypothesize that this kLANA function evolved as a mechanism to limit immune detection by rapidly enforcing latency.

We previously demonstrated that kLANA, but not mLANA, could repress the promoter for the MHV68 *ORF50*/RTA gene in a manner that correlated with reduced *Orf50* transcription during infection (12), an observation that highlights a functional difference in these two LANA homologs. It is important to note that the transcription profile of KLKI MHV68 appears to reflect a repressive phenotype, and not just the absence of an mLANA-specific function, since mLANA-null MHV68 exhibited progressive amplification of viral gene expression over time, whereas KLKI MHV68 did not. It is also notable that repressed transcription during KLKI virus infections was overcome by expression of mRTA *in trans*, which further supports the interpretation that kLANA mediates repression of lytic MHV68 transcription. Altogether, these data support the conclusion that kLANA-directed curtailment of viral transcription and replication skews KLKI MHV68 toward a more pro-latency phenotype, rather than undergoing the vigorous lytic replication exhibited by the WT virus.

By extending our observations to KSHV and humans, we speculate that repression of lytic replication by kLANA enables primary KSHV infections to be minimally immune-stimulating, hypothetically limiting both viral antigen production and infection-associated inflammation. Indeed, after *de novo* infection of cells in culture, KSHV lytic gene transcription is transient, typically shutting off within ~24 h, and infectious virus particles are usually not detected (11, 45). Taken together with transcriptional analyses of KLKI MHV68 infection *in vitro*, the observation that adaptive immune cell activation and splenomegaly are reduced relative to mice infected with the WT virus indicates that KLKI MHV68 infection is less immunostimulatory *in vivo*. Moreover, the absence of the inflammation-driven IM-like disease splenomegaly implicates kLANA in dampening infection-associated pathologies.

To elaborate further on this idea, primary infection of adolescent and adult humans by the related GHV, EBV, can be symptomatic, manifesting as IM, a pathology driven by inflammatory CD4^+^ and CD8^+^ T cells (46). The IM-like syndrome caused by MHV68 in mice also correlates with potent Th1-type virus-specific adaptive immunity after primary infection (47), which is not induced by KLKI infections. Ample evidence demonstrates that MHV68 and EBV benefit from the polyclonal immune response, enhancing the establishment of latency. However, unlike EBV and MHV68, there are no known clinical symptoms associated with primary KSHV infection regardless of age of acquisition (48, 49). While other viral and host mechanisms also likely contribute, this highlights a putative role for kLANA in minimizing processes that drive immune stimulation during primary infection *in vivo,* suggesting that KSHV employs a “below the radar” approach to colonize its hosts despite utilizing similar routes of transmission.

Since suppression of the lytic cycle correlated with reduced adaptive immune stimulation by KLKI MHV68, we next sought to determine if increasing the amount of viral antigens would overcome kLANA-mediated impacts on immune activation, starting by simply testing the dose response of the inoculating virus. The minimum infectious dose for MHV68 to establish latency in a mouse is less than a single PFU of the virus (33); however, the impact of the inoculating dose on adaptive immunity to MHV68 has not, to our knowledge, been tested previously. In fact, how the inoculating dose influences adaptive immunity against most viral infections in general is only minimally explored. We found that both B cell and T cell activation remained low for all doses of KLKI MHV68 tested relative to the WT virus. Only virus-specific antibody production appeared to correlate with the inoculating dose, which seems to agree with vaccination studies using the non-replicating virus (18). Given that the inoculating dose had no effect on KLKI MHV68 latency establishment, our data suggest that a kLANA-intrinsic function, and not simply the amount of input virions, reduces immune cell activation during infection.

Several possibilities could underlie the observation that high-dose KLKI MHV68 infection is less immunogenic: (i) despite a higher inoculating dose of antigen, the decreased production of lytic viral antigens within cells results in less antigen presentation for the host to detect; (ii) fewer danger-associated molecular patterns are generated by KLKI MHV68 infection to elicit potent adaptive activation; or (iii) kLANA modulates host-cell signaling pathways to prevent detection and/or activation by immune cells. These hypotheses, which are not mutually exclusive, could be tested in future experiments.

Since high-dose infections did not elicit strong immune activation, we next tested a model with enhanced viral replication to attempt to overcome kLANA-mediated dampening of adaptive immunity. IFNs act as critical mediators of host restriction during GHV infection, limiting acute replication and reactivation (34). Furthermore, IFNα/β levels remain elevated during chronic GHV infection, implying that type I IFNs also regulate viral latency (50). Since the enhancement of KLKI MHV68 lytic viral replication/reactivation in *Ifnar1^−/−^* mice led to increased adaptive immune stimulation and splenomegaly, we postulate that the absence of type I IFN signaling enables KLKI infections to surpass an antigenic and/or danger signaling threshold necessary to engage the adaptive immune response. These data suggest the existence of a trigger point, and once GHV lytic replication amplifies beyond this point during infection, potent adaptive immune responses are elicited.

It is important to consider the possibility that a kLANA-mediated role in antagonizing IFN-related signaling is not functional in mouse cells. If so, this would render KLKI MHV68 more susceptible to IFN-mediated shutdown of viral replication. However, we think this is unlikely, given that mLANA-null MHV68 does not exhibit the same repressed lytic gene expression profile as the kLANA knock-in virus. This, paired with direct repression of the *ORF50*/RTA promoter by kLANA (12), suggests that suppressed lytic replication during KLKI infection reflects a kLANA-specific function not encoded by mLANA rather than increased sensitivity to the effects of IFN. It is tempting to speculate that kLANA works in concert with IFNα/β-signaling to suppress viral transcription and lytic replication, perhaps integrating with STAT1 signaling to repress MHV68 transcription. Notably, kLANA is present on STAT1-responsive promoters in latently infected PEL cells (42, 51), but whether this occurs during KLKI infection of murine cells remains to be determined. Regardless of the distinct mechanism, it is clear that enhanced lytic viral replication/reactivation during infections of *Ifnar1^−/−^* mice is sufficient to potently stimulate adaptive immune cell activation irrespective of whether kLANA mediates the repression of mRTA in this setting.

In essence, our data suggest that the “stealth” approach to infection adopted by KSHV, in part mediated by kLANA, likely enables viral colonization of the host while remaining below the triggering threshold that sounds the alarm for immune cell activation during primary infection. In agreement with this idea, it is interesting to note that KSHV seroconversion occurs several months after viral genomes are detectable in peripheral blood of newly infected individuals (52). By coupling low-level lytic replication at minimal infectious doses, a virus that is “pro-latent,” like KLKI MHV68 or KSHV, may evade eliciting potent antiviral adaptive immune responses after primary infection, while still efficiently colonizing a host.

Our work highlights an important aspect of studying KSHV-MHV68 chimeras to assess how viral factors affect GHV pathogenesis and development of host antiviral immunity. In general, information regarding how KSHV adaptive immune responses develop after primary infection in humans is lacking. Utilizing GHV chimeras like KLKI MHV68 provides an opportunity to elucidate mechanisms underlying KSHV pathogenesis in a living organism. Since our work demonstrates that KLKI MHV68 efficiently establishes latent infection while minimally stimulating adaptive immunity, we propose that kLANA-mediated repression of lytic viral replication similarly contributes to KSHV immune evasion in humans. Since humans can be infected simultaneously with multiple strains of KSHV (53, 54) and KSHV shows evidence of intra-strain recombination (55, 56), the absence of potent antiviral immunity could enable these events to occur, facilitating KSHV adaptation and evolution.

## MATERIALS AND METHODS

### Mice and infections

Male and female C57BL/6 or *Ifnar1^−/−^* (B6(Cg)-Ifnar1tm1.2Ees/J) mice were purchased from Jackson Laboratories or were bred and maintained in-house in sterile conditions; 6- to 12-week-old mice were anesthetized using isoflurane and inoculated with the indicated dose of the virus diluted in antibiotic-free DMEM (20 μL) for intranasal inoculations. Lungs, spleens, and sera were harvested at the indicated times postinfection, as described previously (57).

### Cells and viruses

Vero-cre cells (58), 3T12 fibroblasts (ATCC CCL-164), baby hamster kidney fibroblasts (BHK21) (ATCC CCL-10), and HEK 293T cells (ATCC CRL-3216) were cultured in Dulbecco’s modified Eagle medium (DMEM) supplemented with 10% bovine calf serum (BCS), 100 U/mL penicillin, 100 μg/mL streptomycin, and 2 mM L-glutamine. Cells were cultured at 37°C with 5% CO_2_ and ~99% humidity. Viruses used in this study were described previously (12, 21).

### Cell culture infections

3T12 fibroblasts were infected with the indicated viruses and MOIs. Briefly, viral stocks were diluted in low-volume (200 μL) of DMEM and added directly onto fibroblast cell monolayers seeded the previous day at a density of 2 × 10^5^ cells/well in six-well plates. Plates were incubated at 37°C and rocked gently every 15 min for 1 h. After 1 h of adsorption, the cells were incubated in an appropriate culture volume of cDMEM for the indicated times at 37°C. For multistep growth curves, infected cells were frozen at −80°C at the indicated time points. Cells were subjected to freeze-thaw lysis to release progeny virions, and the lysates were serially diluted for plaque assays as described (59).

### Plaque assays

Plaque assays were performed as previously described (59) using BHK21 cells. Briefly, infected cells were overlaid with 1.5% methylcellulose in DMEM supplemented with 2.5% calf serum, 100 U/mL penicillin, 100 μg/mL streptomycin, and 2 mM l-glutamine and incubated at 37°C for 4–6 days. Cell monolayers were stained with a solution of crystal violet in formalin for the identification and quantification of plaques.

### CRISPR-Cas9 deletion of *Ifnar1*

Ifnar1 was deleted in 3T12 fibroblasts using the CRISPR-Cas9 system. The pLentiCRISPRV.2 (Addgene #52961) plasmid was digested with BsmBI-v2 and ligated with guide RNA sequences specific for Ifnar1 in Table 3 (60). Cloned plasmids were transfected into HEK293T cells in combination with psPAX2 and pMD2.G plasmids using polyethyleneimine (Sigma). Supernatants were collected 72 h post-transfection, passed through 0.45 μm syringe filters, and applied to 3T12 fibroblast in 6-well plates. 3T12 fibroblasts were transduced with retroviral supernatants by centrifugation for 30 m at 1,000 × *g* for 30 m at 37°C in the presence of 10 μg/mL polybrene (Santa Cruz Biotechnology). Cells were washed, and media were replaced with cDMEM; 48 h post-transduction, puromycin (Invitrogen; 2 μg/mL) was added to the medium. Puromycin-selected 3T12s were tested for Ifnar1 deletion by treatment with recombinant mouse IFN-β (Cell Signaling Technologies).

**TABLE 3:** Primers for qRT-PCR and guide RNA oligonucleotide sequences for CRISPR/Cas9 genome editing

Gene	Forward primer/oligo sequence	Reverse primer/oligo sequence	Expected size (bp)	
M3	ATTCTTCAGAGATGGCGCCAAG	TCTTATGTGGATGTGGGCATGC	133	
ORF6	CCATGCCATCGTCTTTCACAAC	TGGTCACAGTTTTCATGGGTGC	266	
ORF8	ATTGTAGACATGGTGGCACGC	TCTGGTGGCTGTTTTCCAGG	261	
ORF25	TATAGCGCACGGTGATGGAAGAAG	GATTGGAGAATAAAGGTGGGCG	285	
ORF50	AAAAGTTCTGCATCCCAGACCC	AGGGCTAATGGGTGAAAATGGC	293	
M7	CCACCTCCAATGCAGATGTTTC	ATTTTGGGAAGGGGTGGTTG	128	
ORF9	CAATTGCTGTATCCCATCTGCG	GGAAACCCACATTCACCCAAAC	284	
ORF65	AGACAGGGTCCATCATTTTGGC	TTGGCAAAGACCCAGAAGAAGC	159	
ORF68	CTCAAATACACTGGCCGCCATC	CGTGCTTGAGATATGAGTGAGT	168	
ORF72	TTAGCACTGGGCGTTTCATG	CACGTGGATGTTTTGTGTGTGC	425	
kORF73	TACGGTTGGCGAAGTCACATC	CCTCGCAGCAGACTACACCTCCAC	204	
ORF73	TGTTGTGTGCCAGAAGCTTGTG	TACCAGGCACAACACAACAGT	235	
ORF75c	AAGACACAGAGGCTGGGAGA	GACGGGTAGACGTGGTGACT	157	
ORF75b	CAGCCTCTCAACCTTTCCAG	ATAGGAGCCACCGTTGATTG	155	
M11	AGAAAAGCGGGACTTATTGGGC	TTTTCCAGTTCTTGAGGGCAGG	195	
B_2_M	TTCTGGTGCTTGTCTCACTGA	CAGTATCTTCGGCTTCCCATTC	104	
GAPDH	TGACCTCAACTACATGGTCTACA	CTTCCCATTCTCGGCCTTG	85	
Ifnar1 sgRNA 1	CACCGCTTCTAAACGTACTTCTGGG	AAACCCCAGAAGTACGTTTAGAAGC		
Ifnar1 sgRNA 2	CACCGTCTCCATGTACAAGCCTCAG	AAACCTGAGGCTTGTACATGGAGAC		
mSAFE 7500 sgRNA	CACCGAAGTGGTCTAGTTAACTA	AAACTAGTTAACTAGACCACTTC		

### Splenocyte isolation and limiting-dilution assays

Spleens were homogenized through a sterile 40-μm filter, and red blood cells were lysed by incubating tissue homogenate in 8.3 g/L ammonium chloride for 10 min at room temperature with shaking. Frequencies of cells harboring MHV68 genomes were determined using limiting-dilution (LD) PCR analysis with primers specific for an ORF50 locus as previously described (61). Frequencies of latently infected cells capable of undergoing reactivation were determined using a limiting-dilution analysis for cytopathic effect on an indicator monolayer as previously described (18, 62), but using BHK21 cells in place of murine fibroblasts to shorten the length of the assay. Cytopathic effect was scored 5–7 days post-plating. Side-by-side comparisons of reactivation efficiency on BHK21 and MEFs were performed prior to instituting this change.

### Immunofluorescence microscopy

3T12 fibroblast cells were plated infected as described above. Infected cells were washed with cold phosphate-buffered saline (PBS) and fixed in 10% phosphate-buffered formalin. Cells were permeabilized for 15 min with 0.5% Triton X-100-Tris-buffered saline (TBS) at room temperature. Supernatant was aspirated, and the cells were incubated in blocking buffer (5% bovine serum albumin [BSA] and 0.1% Triton X-100, TBS) for 30 min at room temperature. Primary antibodies diluted in blocking buffer 1:1,000 at 4°C overnight to probe for lytic antigens using serum from infected WT C57BL/6 mice. Samples were washed three times with cold wash buffer (0.1% Triton X-100, TBS). AlexaFluor 488-conjugated goat anti-mouse secondary antibodies specific to primary antibodies diluted in blocking buffer 1:2,500 and were added to the samples for 2 h in the dark at room temperature. Samples were washed three times with wash buffer and incubated with 1 μg/mL DAPI (4′,6-diamidino-2-phenylindole) (Thermo Fisher) at room temperature to stain DNA. Cells were imaged by fluorescence microscopy using ×20 magnification on a Cytation 10 Confocal Imaging Reader (Agilent).

### Immunoblot analyses

Immunoblot analyses were performed as previously described (63). Briefly, cells were lysed with radioimmunoprecipitation (RIPA) buffer (150 mM NaCl, 20 mM Tris, 2 mM EDTA, 1% NP-40, and 0.25% deoxycholate) supplemented with phosphatase and protease inhibitors. Samples were centrifuged at 16,000 × *g* to remove insoluble debris. The protein content for each sample was quantified using a DC protein assay (Bio-Rad). Samples were diluted in 4× Laemmli sample buffer and resolved by sodium dodecyl sulfate-polyacrylamide gel electrophoresis (SDS-PAGE) using 4%–20% Mini-PROTEAN TGX precast gels (Bio-Rad) or 10% polyacrylamide gels prepared in-house. Resolved proteins were transferred to nitrocellulose membranes (Bio-Rad), blocked in either 5% BSA or 5% milk, and blots were probed with the indicated primary antibodies in Table 4. Antibody-bound proteins were recognized using horseradish peroxidase (HRP)-conjugated secondary antibodies (Jackson ImmunoResearch). Chemiluminescent signal was detected using a ChemiDoc MP imaging system (Bio-Rad) on blots treated with ProSignal Pico ECL reagent (Prometheus).

**TABLE 4:** Antibodies used for immunoblot analyses.

Immunogen	Clone	Supplier	Dilution
Rabbit phospho-STAT1 (Tyr701)	58D6	Cell Signaling Technologies	1:2,000
Rabbit anti-STAT1	Polyclonal	Cell Signaling Technologies	1:2,000
Mouse anti-FLAG	M2	Millipore Sigma	1:1,000
Rabbit anti-mLANA	Polyclonal	In-house	1:1,000
Rat anti-kLANA (HHV-8 LNA1)	LN53	Millipore Sigma	1:1,000
Mouse anti-beta tubulin	4G5	Millipore Sigma	1:2,000
Mouse anti-beta actin	W16197A	BioLegend	1:2,000
MHV68 lytic antigens (42-dpi mouse antiserum)	Polyclonal	In-house	1:1000

### RNA isolation and qRT-PCR

Total RNA was extracted from homogenized tissue or cells using a Quick-RNA Miniprep Kit (Zymo Research) according to the manufacturer’s instructions. cDNA was synthesized using the iScript cDNA Synthesis Kit (Bio-Rad) according to the manufacturer’s instructions. For qRT-PCR, cDNA was diluted 1:3 and reverse transcription-PCR (RT-PCR) for *ORF50*, *ORF6*, *ORF9*, *ORF68*, *ORF25*, *ORF75c*, *ORF75b*, *ORF8*, *M7*, *M3*, *M11*, *ORF72*, *mORF73*, *kORF73, Actin*, and *B2m* transcripts was performed using SsoAdvanced Universal SYBR Green Supermix (Bio-Rad) using inner primers described in Table 3 (64). Cycling conditions were 40 cycles of 94°C for 1 min, 62°C for 1 min, and 72°C for 1 min in Applied Biosystems StepOnePlus PCR system.

**TABLE 5:** Antibodies used for flow cytometry analyses

Surface marker	Clone	Fluorophore	Dilution	Supplier
Anti-mouse B220	RA3-6B2	rF710/PerCP-CY5.5	1:300	TONBO
Anti-mouse CD19	GD5	BV650	1:300	BioLegend
Anti-mouse GL7	GL7	APC	1:300	BioLegend
Anti-mouse CD38	90	PE-Cy7	1:300	BioLegend
Anti-mouse CD138	281-2	PE	1:400	BioLegend
Anti-mouse CD3e	145.2C11	BV510/PerCP-Cy5.5	1:300	TONBO
Anti-mouse CD11b	M1/70	PerCP-Cy5.5	1:300	TONBO
Anti-mouse CD11c	N418	PerCP-Cy5.5	1:300	TONBO
Anti-mouse TER-119	TER-119	PerCP-Cy5.5	1:300	TONBO
Anti-mouse CD4	RM4-5	rF710	1:300	TONBO
Anti-mouse CD8a	2.43	vF450	1:300	TONBO
Anti-mouse CD44	IM7	PE, BV650	1:400	BioLegend
Anti-mouse CD62L	MEL14	BV605	1:300	BioLegend
Anti-mouse PD-1	J43	PE-Cy7	1:300	BioLegend
Anti-mouse CXCR5	L138B7	BV711	1:300	BioLegend
Anti-mouse IFNAR1	MAR1-5A3	PE	1:100	BioLegend
Anti-mouse IFNγ	XMG-1.2	PE-Cy7	1:100	BioLegend

### Flow cytometry analysis and antibodies

In total, 3 × 10^6^ spleen cells were resuspended in 100 μL of FACS buffer (1× PBS, 2% BSA, and 5 mM EDTA) and blocked with anti-CD16/CD32 (BD Biosciences) for 15 min on ice. Cells were incubated on ice for 30 min with specific antibodies to identify surface markers. Lymphocyte subsets were identified with antibodies against indicated surface markers seen in Table 5. The data were collected on a BD LSR Fortessa, BD FACS Symphony (Beckman Coulter), or Cytek Northern Lights (Cytek Biosciences) flow cytometer and analyzed using FlowJoX v10.0.7 (Treestar Inc., Ashland, OR). Cells were first gated on lymphocytes based on forward and side scatter parameters, and live cells were identified by exclusion of Fixable Viability dye eFlour 780 (eBiosciences) prior to subgating.

### Intracellular cytokine staining

In total, 5 × 10^6^ spleen cells were resuspended in RPMI supplemented with 50 μM 2-mercaptoethanol, 1 mM sodium pyruvate, 10% fetal calf serum (FCS), 100 U/mL penicillin, 100 μg/mL streptomycin, and 2 mM L-glutamine. Cells were restimulated with phorbol 12-myristate 13-acetate (PMA; 50 ng/mL) and ionomycin (500 ng/mL) and cultured at 37°C with 5% CO_2_ and ~99% humidity for 5 h prior to surface staining. For intracellular cytokine staining, the BD Cytofix/CytoPerm Kit (BD Biosciences) was used according to the manufacturer’s instructions. Cells were incubated for 30 min with specific antibodies to determine IFNγ production.

### MHV68-specific tetramer staining

H-2D^b^-p56 MHC-peptide complexes were provided as biotinylated monomers by the NIH tetramer core facility and reconstituted with streptavidin-conjugated APC (used at a final dilution of 1:100). Tetramer staining was conducted in tandem with surface staining. Cells were fixed with 4% paraformaldehyde prior to data acquisition and analysis as described above.

### Enzyme-linked immunosorbent assays (ELISAs)

ELISA plates (MidSci) were coated with 0.5% paraformaldehyde-fixed viral antigens diluted 1:20 in carbonate buffer (0.0875 M Na_2_CO_3_, 0.0123 M HCO_3_, pH 9.2) and incubated overnight at 4°C. Plates were washed in PBS with 0.05% Tween-20 (PBS-T) and blocked with 3% powdered milk-PBS-T for 1 h at 37°C. Mouse sera were collected by cardiac puncture after euthanasia, and sera were serially diluted in 1% milk-PBS-T and incubated for 1 h at 37°C. Virus-specific IgG was detected by incubation with HRP-conjugated donkey anti-mouse IgG (dilution 1:5,000; cat# 715-035-150; Jackson ImmunoResearch). SureBlue substrate (KPL) was added for 3–5 min to detect virus-specific antibodies and read at 450 nm on a BioTek Synergy LX plate reader.

### Plaque-reduction neutralization assays

Neutralization was tested by means of a plaque reduction as described (65). Briefly, sera were diluted 1:40 prior to 3-fold serum dilutions in cDMEM. Each dilution was incubated with 25 PFU of MHV68 for 1 h on ice. The virus/serum mixture was then added to a sub-confluent BHK21 monolayer (4 × 10^4^ cells/well) plated the previous day in a 24-well plate, in triplicate. Baseline consisted of the virus standard alone. Infected cells were overlaid with methylcellulose and incubated at 37°C for 3–4 days. The overlay was removed, and the monolayers were stained with a solution of crystal violet (0.1%) in formalin to visualize plaques. Percent neutralization was determined by comparing the number of plaques in experimental wells to no-serum controls.

### Statistical analysis

Data were analyzed using GraphPad Prism software (GraphPad Software, http://www.graphpad.com, La Jolla, CA). Total numbers of immune cells were analyzed by one-way ANOVA followed by post-tests depending on the parametric distribution. Based on Poisson distribution, frequencies of viral genome-positive cells and reactivation were obtained from a nonlinear regression fit of the data, where the regression line intersects 63.2%. Titer data were analyzed with an unpaired *t*-test.

## SUPPLEMENTAL MATERIAL

Supplemental Figures mbio.00297-26-s0001.pdf Fig. S1 to S4.

ASM does not own the copyrights to Supplemental Material that may be linked to, or accessed through, an article. The authors have granted ASM a non-exclusive, world-wide license to publish the Supplemental Material files. Please contact the corresponding author directly for reuse.
