# Supplementary material for: Repression of productive viral replication by KSHV LANA correlates with reduced adaptive immune activation: potential implications for KSHV immune evasion
Source: mBio. 2026 Jul 23;17(8):e00297-26. doi: 10.1128/mbio.00297-26 (PMC13463888; doi:10.1128/mbio.00297-26)
Supplement: Supplementary file 1 [file mbio.00297-26-s0001.pdf]

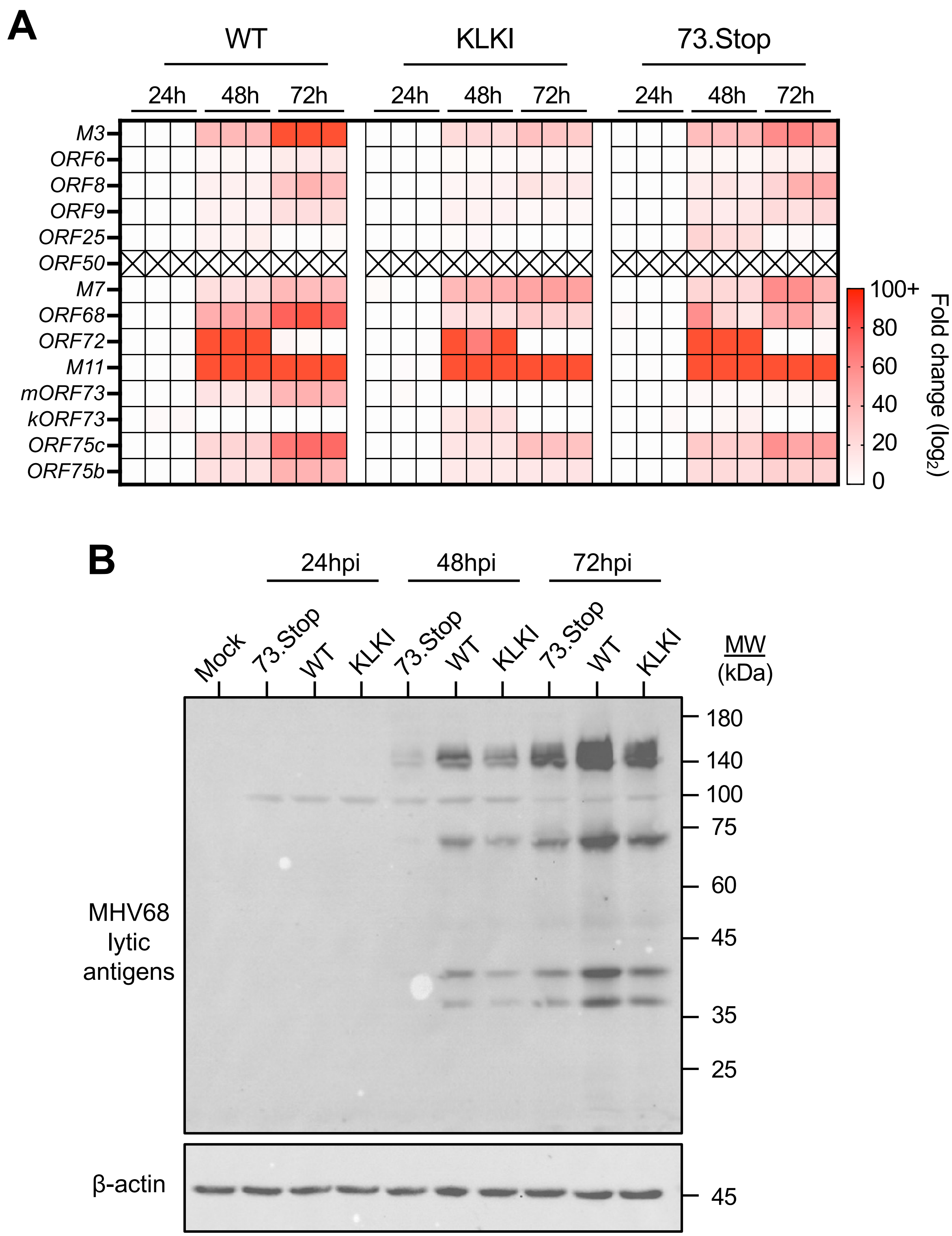

Supplemental Figure 1. Expression of mRTA *in trans* rescues lytic antigen accumulation during KLKI MHV68 infection. RTA-expressing 3T12 fibroblasts were infected with the indicated virus at an MOI of 0.1 PFU/cell. (A) RNA was collected at the indicated times postinfection. Viral transcripts were quantified by qRT-PCR relative to *B2m* using the ΔΔCT method and normalized to transcript abundance at 24h postinfection. (B) Lysates were collected at the indicated times postinfection. Viral proteins were resolved by SDS-PAGE and immunoblot analyses were performed using antibodies that recognize the indicated proteins.

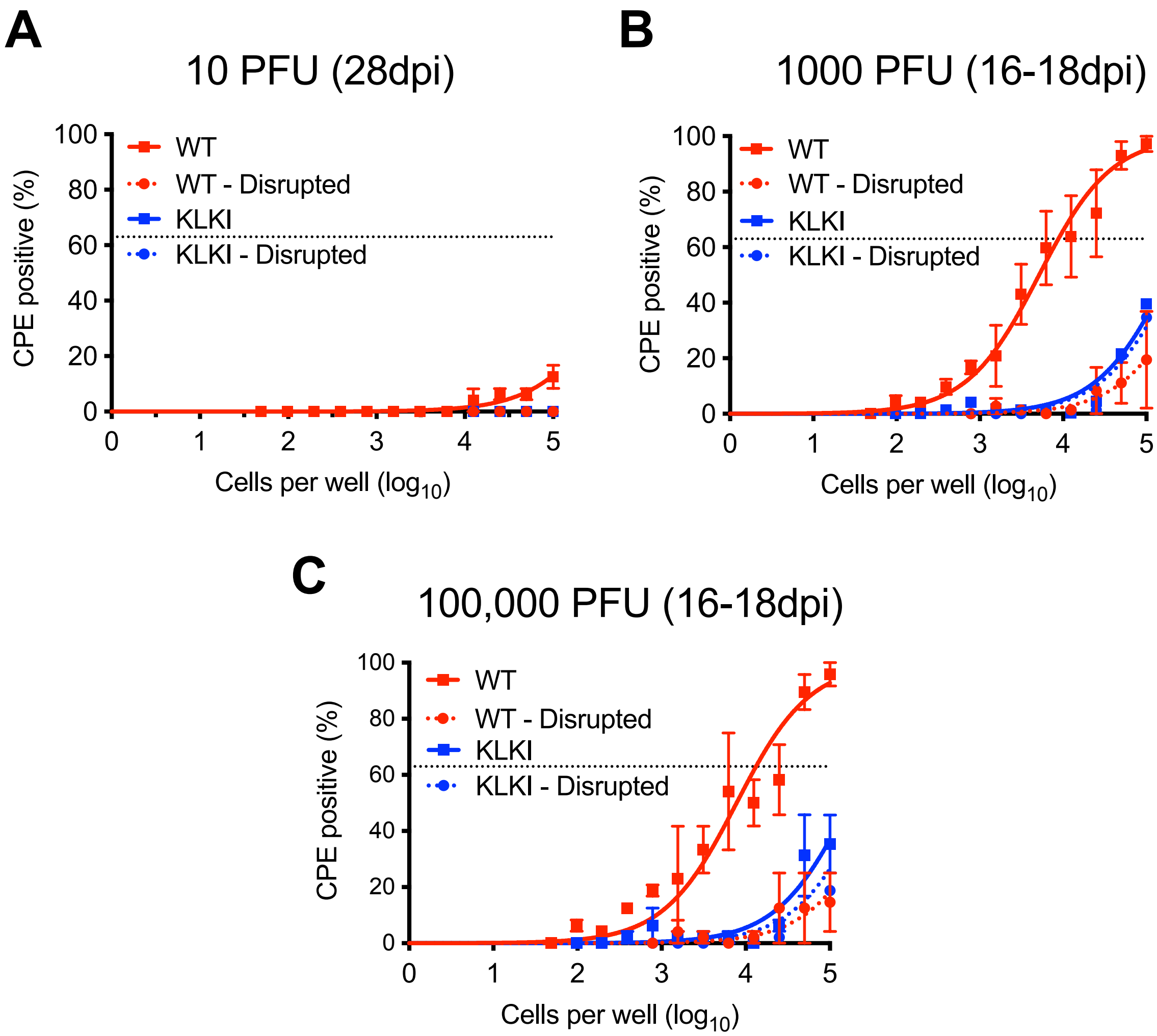

Supplemental Figure 2. Reactivation from latency is reduced after KLKI MHV68 infection. C57BL/6 mice were infected IN with 10, 1000, or 100,0000 PFU of the indicated virus. Mice were sacrificed on day 16-18 postinfection (1000, or 100,000 PFU) or day 28 postinfection (10 PFU). Single-cell suspensions of splenocytes were serially diluted and reactivation frequencies were determined by *ex vivo* plating of splenocytes onto an indicator monolayer. Cytopathic effect was scored one-week postplating. Groups of 3-5 mice were infected and splenocytes were pooled for analysis. Results are means of 2-3 independent infections.

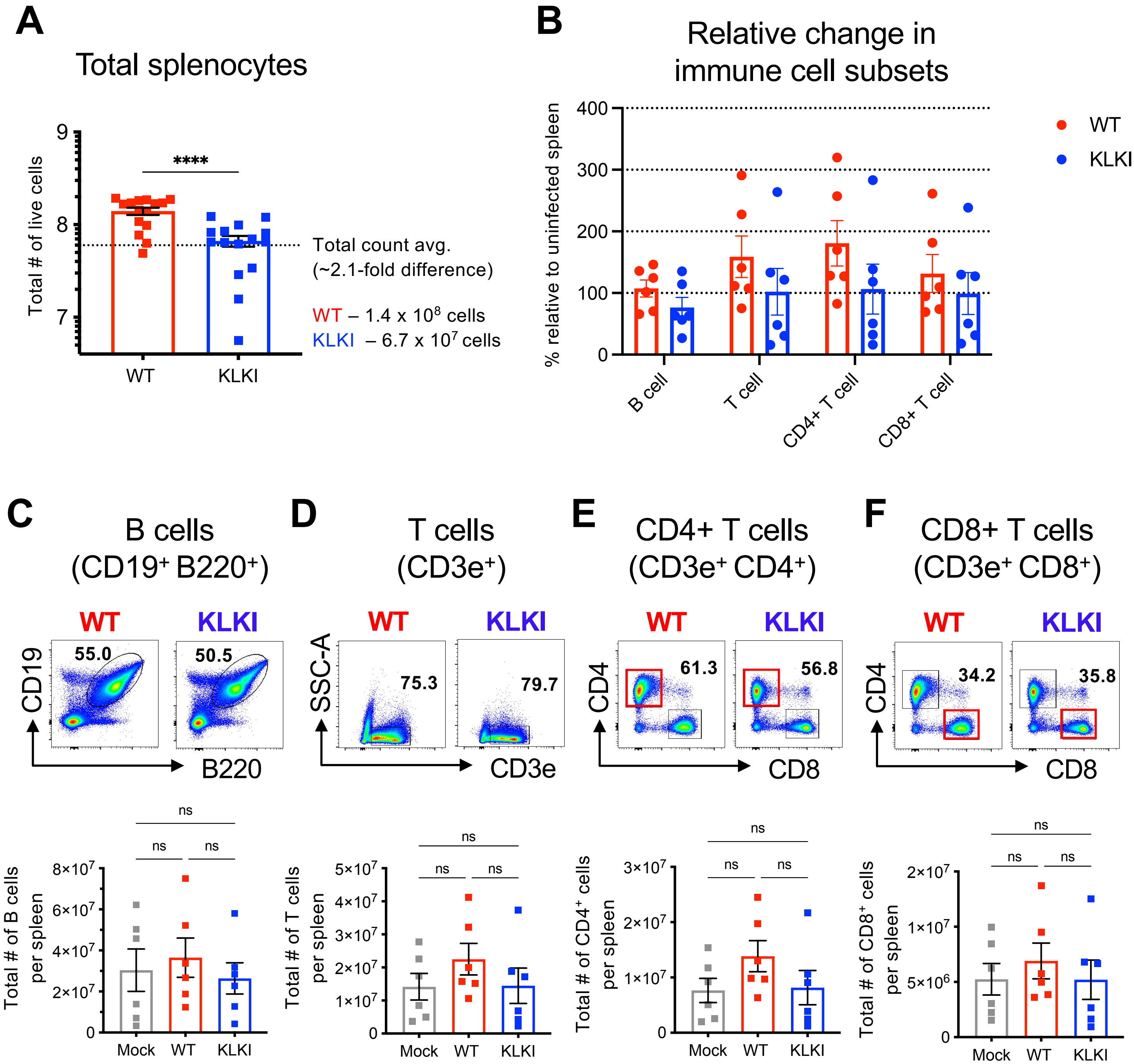

Supplemental Figure 3. **Total B and T cell populations are comparable between WT and KLKI MHV68 infection.** C57BL/6 mice were mock-infected or infected IN with 1000 PFU of the indicated virus. Mice were sacrificed on day 16-18 postinfection. (A) Total spleen cells were quantified. Viable cells were determined by trypan blue exclusion. The dashed line represents the average count for uninfected spleens. (B-F) Cells were analyzed and quantified via flow cytometry. (B) Total numbers of each cell type were determined based on the percentage of total splenocytes isolated. Percentages are relative to mock-infected animals. (C) B cells were defined as $CD19^{+}$/$B220^{+}$, (D) T cells as $CD3e^{+}$, (E) CD4+ T cells as $CD3e^{+}$/CD4+, and (F) CD8+ T cells as $CD3e^{+}$/$CD8^{+}$ cells. Groups of 3-5 mice were used for each infection and analysis. Results are means of 2 independent infections. Each dot represents one mouse. Error bars represent standard error of the mean. ns – not significant. (A) **** denotes $p<0.0001$ in an unpaired Student's *t*-test.

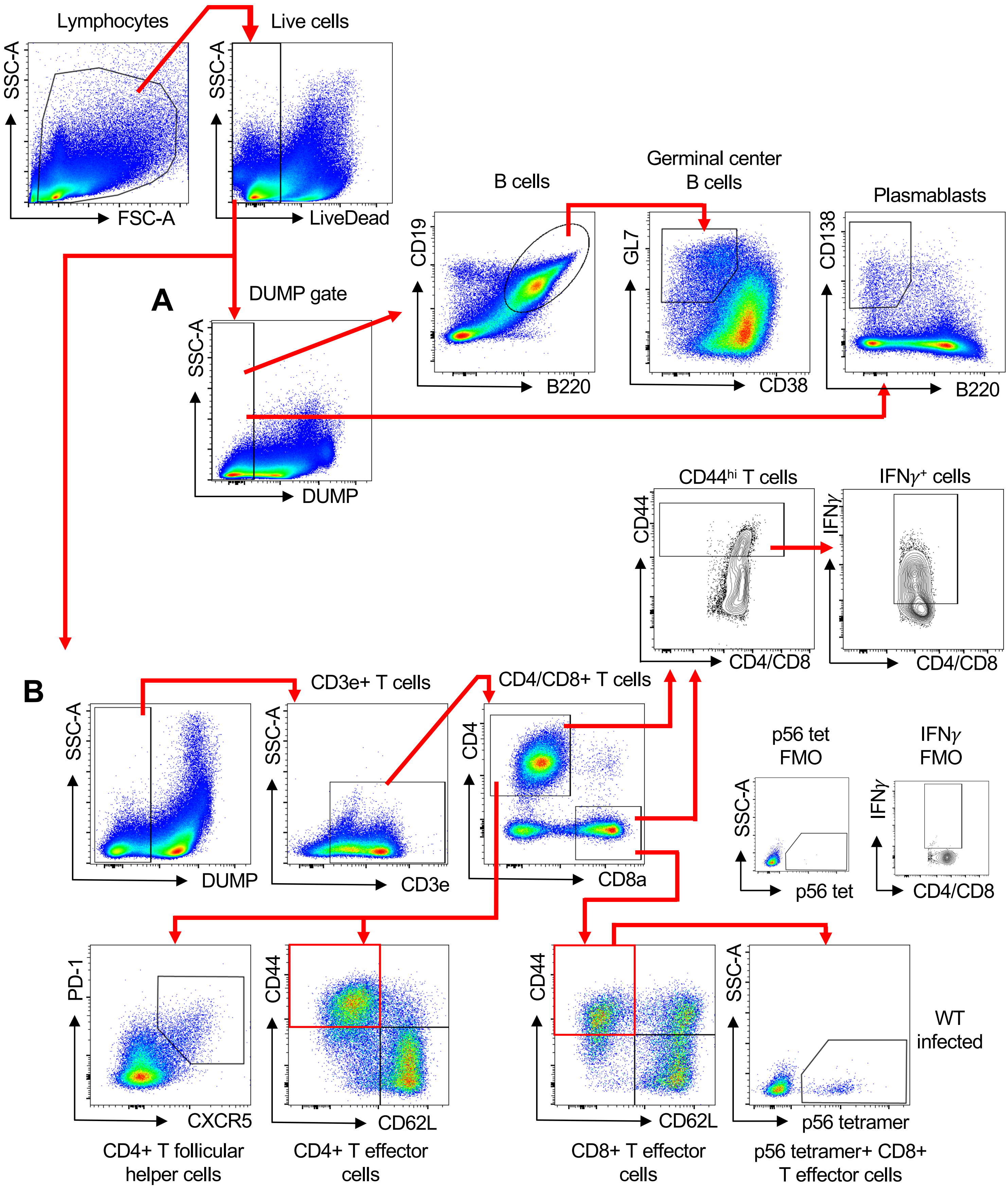

Supplemental Figure 4. **Flow cytometry gating strategies.** C57BL/6 mice were mock-infected or infected IN with 1000 PFU of WT MHV68. Mice were sacrificed on day 16-18 postinfection and spleens were analyzed by flow cytometry. (A-B) Gating strategy for B and T cells populations. (A) For B cell populations, DUMP exclusion gate was defined as viable CD3e+ CD11b+ CD11c+ TER119+ cells. (B) For T cell populations DUMP, exclusion gate was defined as viable B220+ CD11b+ CD11c+ TER119+ cells.
